# A new set of reference housekeeping genes for the normalization RT-qPCR data from the intestine of piglets during weaning

**DOI:** 10.1371/journal.pone.0204583

**Published:** 2018-09-26

**Authors:** Shujin Wang, Binxing Wang, Huan He, Aomin Sun, Chunhua Guo

**Affiliations:** 1 College of Life Science and Technology, Southwest Miznu University, Chengdu, China; 2 Department of Molecular Genetics and Cell Biology, Maastricht University, Maastricht, The Netherlands; 3 Key Laboratory of Qinghai-Tibetan Plateau Animal Genetic Resource Reservation and Utilization, Ministry of Education, Southwest Minzu University, China; INIA, SPAIN

## Abstract

The intestinal mucosal development of piglets (*Sus scrofa*) during the weaning stage is important to their disease susceptibility and later growth. Quantitative real-time PCR (RT-qPCR) is commonly used to screen for differentially expressed genes and, for accurate results, proper reference housekeeping genes are essential. Here we assessed the mRNA expression of 18 well-known candidate reference genes at different parts of the gastrointestinal tract (GIT) of piglets during the weaning process by RT-qPCR assay. GeNorm analysis revealed that *B2M/HMBS/HPRT1* were the three most stable reference genes and *GAPDH* was the least stable gene in the duodenum, jejunum, ileum, colon, and whole GIT. BestKeeper analysis found that *B2M/HMBS/PGK11*, *HMBS/B2M/HPRT1*, *B2M/HMBS/HSPCB*, *B2M/HPRT1/HMBS*, and *B2M/HMBS/HPRT1* were the most stable genes in the duodenum, jejunum, ileum, colon, and whole GIT, respectively, whereas *GAPDH*, *B-actin*, and *18S rRNA* were the least stable genes at different parts of the GIT. To confirm the crucial role of appropriate housekeeping genes in obtaining reliable results, we analyzed the expression of *ALP* using each of the 18 reference genes to normalize the RT-qPCR data. We found that the expression levels of *ALP* normalized using the most stable reference genes (*B2M/HMBS/HPRT1*) differed greatly from the expression levels obtained when the data were normalized using the least stable genes (*GAPDH*, *B-actin*, and *18S*). We concluded that *B2M/HMBS/HPRT1* were the optimal reference genes for gene expression analysis by RT-qPCR in the intestinal mucosal development stages of piglets at weaning. Our findings provide a set of porcine housekeeping reference genes for studies of mRNA expression in different parts of the pig intestine.

## Introduction

The weaning process is a widespread health concern in the swine industry [1,2] because the rapid dietary shift from sow milk to solid diet contributes to dramatic shifts in the intestinal structure and function and can lead to post-weaning crypt elongation and villous atrophy [3]. The developing intestinal mucosa, which is the first line of defense against a hostile environment within the intestinal lumen [4], is particularly vulnerable to disruptions during the transformation from lactation to weaning and post-weaning that can have long-term impacts on disease susceptibility of mammals [1]. Therefore, understanding the underlying mechanisms associated with intestinal mucosal development is of great importance to later growth [5].

Gene expression analysis, especially by Quantitative real-time PCR (RT-qPCR), has already provided insights into the potential mechanisms involved in the intestinal mucosal development in pig [6,7]. Unlike other methods used in gene expression studies, RT-qPCR data are generally normalized against a set of housekeeping reference genes to minimize the differences among starting materials [8]. Of particular note is the evidence from Huggett et al. (2005), which indicated inappropriate normalization of RT-qPCR data resulted in incorrect conclusions [9]. Thus, the identification of an appropriate set of housekeeping genes as endogenous reference genes is crucial to normalize RT-qPCR data [10,11]. Ideal housekeeping genes are expected to be expressed at a constant level in different tissues at all developmental stages, be unaffected by experimental treatments, and should pass through the same steps involved in gene analysis for qualification [9]. However, several commonly used housekeeping genes, such as *B-actin* (beta-actin), *GAPDH* (glyceraldehyde-3-phosphate dehydrogenase), and *18S* (18S ribosomal RNA), are context-dependent because their expression levels may vary significantly with the experimental conditions being investigated [12], and no set of housekeeping genes appear to be generally applicable for all circumstances. Consequently, the validity of candidate housekeeping genes should be well assessed to circumvent problems for data normalization [11]. To date, information about constant housekeeping genes in the intestinal mucosa of piglets in early life is largely lacking.

In this study, we analyzed the mRNA expression of 18 commonly used housekeeping genes in the duodenum, jejunum, ileum, and colon of piglets at 0, 7, 14, and 21 days post-weaning (dpw) to identify suitable reference genes for RT-qPCR assays. The 18 reference genes were *5S* (5S ribosomal RNA), *RPL4* (ribosomal protein L4), *CANX* (calnexin), *ALDOA* (aldolase A), *PPARGC1A* (PPARG coactivator 1 alpha), *HSPCB* (heat shock 90 kD protein 1, beta), *TBP* (TATA box binding protein), *B2M* (beta-2-microglobulin), *HMBS* (hydroxymethylbilane synthase), *HPRT1* (hypoxanthine phosphoribosyltransferase 1), *B-actin* (beta-actin), *18S* (18S ribosomal RNA), *YWHAZ* (3-monooxygenase/tryptophan 5-monooxygenase activation protein, zeta), *RPL32* (ribosomal protein L32), *PGK1* (phosphoglycerate kinase 1), *PPIA* (peptidylprolyl isomerase A), *TOP2B* (topoisomerase II beta), and *GAPDH* (glyceraldehyde-3-phosphate dehydrogenase).

## Materials and methods

### Animal experiments

The animal procedures used in this study were according to the guidelines of the China Animal Protection Association, and all of the work was approved by the Shenzhen Animal Care Committee.

### Piglets and feeding

A total of 32 conventionally-raised Landrace-Duroc piglets (*Sus scrofa*) weaned at day 26 at a 300-sow batch-farrowing facility (Shenzhen Premix Inve Nutrition Co., Ltd, Shenzhen, China) were used in this study. The randomly selected piglets had an average initial body weight of 6.38 kg and were healthy with no clinical signs of disease. The piglets were assigned randomly to four pens of eight piglets and fed a diet, formulated as a powder, without any in-feed antibiotics based on the guidelines of the National Research Council [13]. All piglets had *ad libitum* access to feed and water. The given and remaining feed was weighted daily at 0800 h.

### Sample collection and processing

One piglet (close to the average body weight in each pen) was chosen from each pen at each time point (i.e., 0, 7, 14, and 21 dpw) and sacrificed with sodium pentobarbital (150.00 mg/kg body weight) by intravenous injection. Then, the whole intestine encompassing duodenum, jejunum, ileum, and colon were dissected out. After slitting the intestine lengthwise and gently rinsing with ice-cold 1×PBS, the mucosa was scraped separately from the duodenum, jejunum, ileum, and colon with a glass slide and the samples were immediately placed in liquid nitrogen for further analysis.

### RNA preparation and RT-qPCR

The RNA preparation and RT-qPCR protocols were as described previously [11]. Briefly, total RNA was extracted from all the samples with Trizol reagent (Invitrogen, Carlsbad, CA, USA). The concentration of the extracted RNA was measured in a NanoDrop ND-1000 spectrophotometer (Agilent Technologies, Palo Alto, CA, USA). The purity of the RNA was measured based on the A260:A230 and A260:A280 ratios, and the quality of RNA was analyzed by 2% agarose gel electrophoresis.

The A260:A230 and A260:A280 ratios ranged from 2.00 to 2.10 and 1.90 to 2.05, respectively, suggesting that all the RNA samples were of high quality. To synthesize the cDNA, 1.00 μg of total RNA from each sample was reverse transcribed using a PrimeScript™ RT Reagent Kit with a gDNA Eraser (Takara Bio Inc., Otsu, Japan), according to the manufacturer’s protocol.

### RT-qPCR assays

The RT-qPCR reactions were carried out in a 96-well plate using a Bio-Rad CFX96TM Real-time PCR Detection System (Bio-Rad, California, USA). The RT-qPCR reaction protocol was as described previously [7]. Briefly, the RT-qPCR reactions were performed in a final volume of 15.0 μl, containing 7.5 μl of 2×SYBR Premix EX Taq II, 5.5 μl of DNase (deoxyribonuclease)/RNase (ribonuclease)-free water, 1 μl of cDNA, and 500 nM of each primer. The PCR cycling program was 95°C (5 min), followed by 40 cycles of 95°C (10 s), 60°C (20 s), and 72°C (20 s). A melting curve analysis was conducted by heating the samples from 65°C to 95°C along continuous fluorescent acquisition. All the RT-qPCR reactions were carried out in triplicate for each sample.

### Selection of reference genes, a target gene, and primer design

We selected 18 well-known candidate reference genes and one target gene for the RT-qPCR assay. The porcine reference genes were obtained from a literature review and the sequences of the selected genes were obtained from the GenBank database [7,14–16]. The primer pairs were designed using Primer Premier 5 (http://www.premierbiosoft.com/primerdesign/), and details of the primer sequences are provided in Table 1.

**Table 1:** Primers and relative information of the reference and target genes.

Gene symbol	Primer sequence (5'-3')	Amplicon length (bp)	Tm(°C)	Source
YWHAZ	F: ATGCAACCAACACATCCTATC	178	62	Erkens et al. (2006)
	R: GCATTATTAGCGTGCTGTCTT			
UBC	F: TTGAGGGGAGGTTTCTAA	82	58.4	M18159
	R: GAATGCAACAACTTTATTGA			
TBP	F: GATGGACGTTCGGTTTAGG	124	55.9	Erkens et al. (2006)
	R: AGCAGCACAGTACGAGCAA			
RPL32	F: AGCCCAAGATCGTCAAAAAG	165	55	NM_001001636
	R: TGTTGCTCCCATAACCAATG			
RPL19	F: GCTTGCCTCCAGTGTCC	82	62	AF435591
	R: GTTGGCGTTGGCGATTT			
PPIA	F: GGGAGAAAGGATTTGGTTAT	175	62	NM_214353
	R: ATGGACAAGATGCCAGGAC			
PPARGC1A	F: CCTGCATGAGTGTGTGCTCT	107	59	Muráni et al. (2007)
	R: CTCAGAGTCCTGGTTGCACA			
PGK1	F: GGGCTAAGCAGATTGTATG	180	52.8	NM_001099932
	R: TGACTTTATCCTCCGTGTT			
HSPCB	F: GGCAGAAGACAAGGAGAAC	131	55.9	AF288819
	R: CAGACTGGGAGGTATGGTAG			
CANX	F: CAATGATGGATGGGGTCTGAA	135	60	Muráni et al. (2007)
	R: AACACAGGTAATGCCACAGTCAA			
ALDOA	F: GAACCAACGGCGAGACAA	142	55.6	AY359812
	R: ATGATGGCGAGGGAGGAG			
5S	F: GCCCGATCTCGTCTGATCT	93	54	AF329851
	R: AGCCTACAGCACCCGGTATT			
18S	F: CCCACGGAATCGAGAAAGAG	125	53	Wang et al.(2016)
	R: TTGACGGAAGGGCACCA			
B2M	F: CAAGATAGTTAAGTGGGATCG	161	60	Wang et al.(2016)
	R: TGGTAACATCAATACGATTTC			
B-actin	F: GGATGCAGAAGGAGATCACG	134	51	Wang et al.(2016)
	R: ATCTGCTGGAAGGTGGACAG			
GAPDH	F: CCTTCCGTGTCCCTACTGC	195	55.9	AF017079
	R: CATCAAAGGTAGAAGAGTGAGTGTC			
HMBS	F: AGGATGGGCAACTCTACCTG	83	59.5	Wang et al.(2016)
	R: GATGGTGGCCTGCATAGTCT			
HPRT	F: GGACTTGAATCATGTTTGTG	91	59.5	Wang et al.(2016)
	R: CAGATGTTTCCAAACTCAAC			
ALP	F: CTAAAGGGGCAGATGAATGG	105	55.9	Lackeyram et al.(2010)
	R: CACCTGTCTGTCCACGTTGT			

Prior to the RT-qPCR assay, we performed agarose-gel-electrophoresis and conventional PCRs (S1 Fig) to verify the amplified products and assess the gene-specific primers. All the RT-qPCR products were sequenced and aligned against the pig genome using BLAST (https://blast.ncbi.nlm.nih.gov/Blast.cgi) to validate their identity. Primer specificity was further assessed by melting curve analysis. Only a single band of the expected size should be observed for each of the primers and the sequences should exactly match the GenBank sequences. Further, the dissociation step of the melting curve analysis should produce a single unique peak, indicating the primers are suitable for use in RT-qPCR assays.

### Data analysis

We separately pooled the RNA samples collected from the four parts of the intestine at the different growth stages, and then pooled together the RNA samples from all four parts of the intestine at the different growth stages to assess the whole gastrointestinal tract (GIT). Subsequently, the threshold cycle (CT) values of the 18 references genes were analyzed by two-way ANOVA using the GLM procedure in the SAS software (SAS Institute Inc., Cary, NC, USA). The model included the main effects of the different parts of the GIT, growth stages, and their interaction.

We also used two classic software programs, GeNorm (Version 3.4) [17] and BestKeeper (Version 1) [18], to evaluate the 18 candidate reference genes. The GeNorm algorithm defines the standard deviation (SD) of the mRNA expression ratio of reference genes as a pairwise variation under the assumption that the ratio of the mRNA expression is identical across all chosen samples and the tested genes are not co-regulated [11]. A gene-stability measure (M value) is then calculated as the average pairwise variation of a specific reference gene compared with other reference genes. The reference gene with the highest M value is considered the least stable gene. Then, all candidate reference genes are ranked by the stepwise elimination of reference genes with the highest M values. GeNorm can also be used to assess the number of reference genes that are required for dependable normalization.

Unlike GeNorm, BestKeeper uses raw data instead of the relative mRNA expression levels [11]. BestKeeper estimates reference gene stability according to the SD values of each gene and generates an index that is analyzed based on the geometric mean of the CT values. The most stable reference gene is the one that has the highest Pearson coefficients of correlation with the BestKeeper index as well as the lowest SD values.

To further assess the efficacy of the selected reference genes for normalization, the relative mRNA expression of a selected target gene (*ALP*) was calculated during the piglet weaning stages using a 2^−ΔΔCT^ method where ΔΔCT = [(CT_*ALP*_−CT_Reference gene_)_Post weaning-day x_−(CT_*ALP*_−CT_Reference gene_)_Post weaning-day 7_] [19].

Normalization was performed with the 18 references genes and the relative mRNA expression of *ALP* at each part of the GIT was analyzed using the one-factor ANOVA program with the different growth stages as the one factor. The significant results obtained by one-way ANOVA were used in a Duncan’s multiple-range test for multiple-comparisons. The data were expressed as mean ± standard error of the mean (SEM), and differences were considered significant at *P* < 0.05.

## Results

### Primer validation

The amplification efficiency of all the primer pairs varied from 90% to 103% and the coefficient of determination (R^2^) ranged between 0.998 and 1.000 (S2 Fig). Single peaks were observed for the products of all the primer pairs according to the melting curve analysis, and the sequences of the amplified DNA fragments (S1 Table) matched the sequences of the reference and target genes in GenBank (Table 2).

**Table 2:** Sequencing results of genes using BLASTN from NCBI against nucleotide collection.

Gene Name	Best hit in NCBI	Identity
YWHAZ	Sus scrofa 3-monooxygenase/tryptophan 5-monooxygenase activation protein, zeta (YWHAZ), transcript variant X1, mRNA	100%
UBC	Sus scrofa ubiquitin C (UBC), transcript variant X3, mRNA	98%
TBP	PREDICTED: Sus scrofa TATA box binding protein (TBP), mRNA	95%
RPL32	Sus scrofa ribosomal protein L32 (RPL32), mRNA	97%
RPL19	Sus scrofa ribosomal protein L19 (RPL19), mRNA	96%
PPIA	PREDICTED: Sus scrofa peptidylprolyl isomerase A (cyclophilin A) (PPIA), transcript variant X2, mRNA	99%
PPARGC1A	Sus scrofa PPARG coactivator 1 alpha (PPARGC1A), mRNA	97%
PGK1	Phosphoglycerate kinase 1 [Sus scrofa]	100%
HSPCB	Sus scrofa heat shock 90kD protein 1, beta (HSPCB) Mrna	96%
CANX	Sus scrofa calnexin (CANX), mRNA	98%
ALDOA	Fructose-bisphosphate aldolase A [Sus scrofa]	96%
5S	Human 5S ribosomal RNA gene	98%
18S	Sus scrofa 18S ribosomal RNA (RN18S), ribosomal RNA	99%
B2M	Sus scrofa beta-2-microglobulin (B2M), mRNA	99%
B-actin	Sus scrofa actin, beta (ACTB), mRNA	98%
GAPDH	Sus scrofa glyceraldehyde-3-phosphate dehydrogenase (GAPDH), mRNA	97%
HMBS	Sus scrofa hydroxymethylbilane synthase (HMBS), mRNA	96%
HPRT1	Sus scrofa hypoxanthine phosphoribosyltransferase 1 (HPRT1), mRNA	100%
ALP	Sus scrofa alkaline phosphatase, intestinal (ALPI), transcript variant 2, mRNA	99%

### Expression profiling of the 18 candidate reference genes

**Fig 1:**
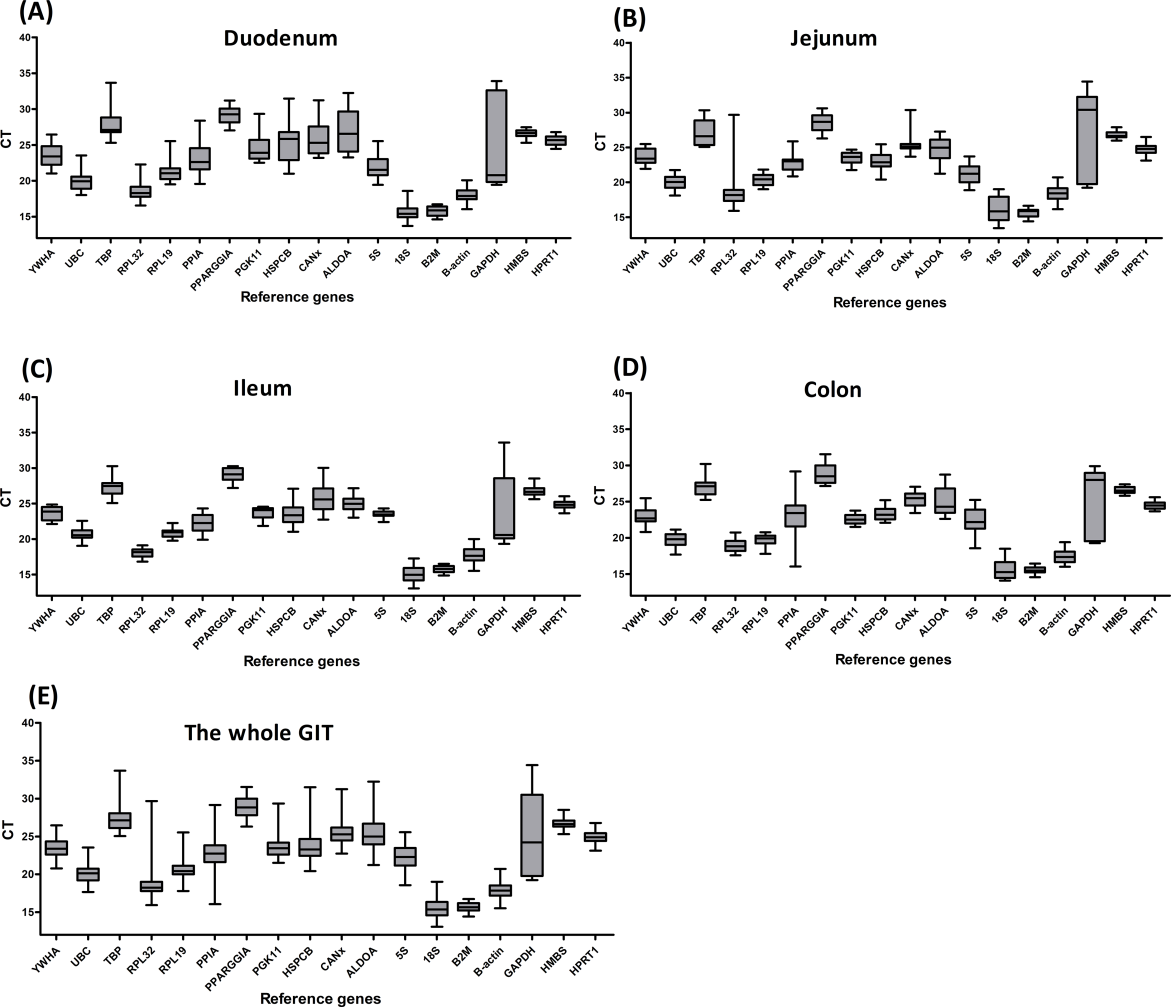
**Distribution of the CT values of the reference genes in the duodenum (A), jejunum (B), ileum (C), colon (D) and whole gastrointestinal tract (E) of piglets.** The boxes encompass the 25th to the 75th percentiles. The middle line in a box marks the median. Whisker caps indicate the minimum and maximum values.

**Fig 2:**
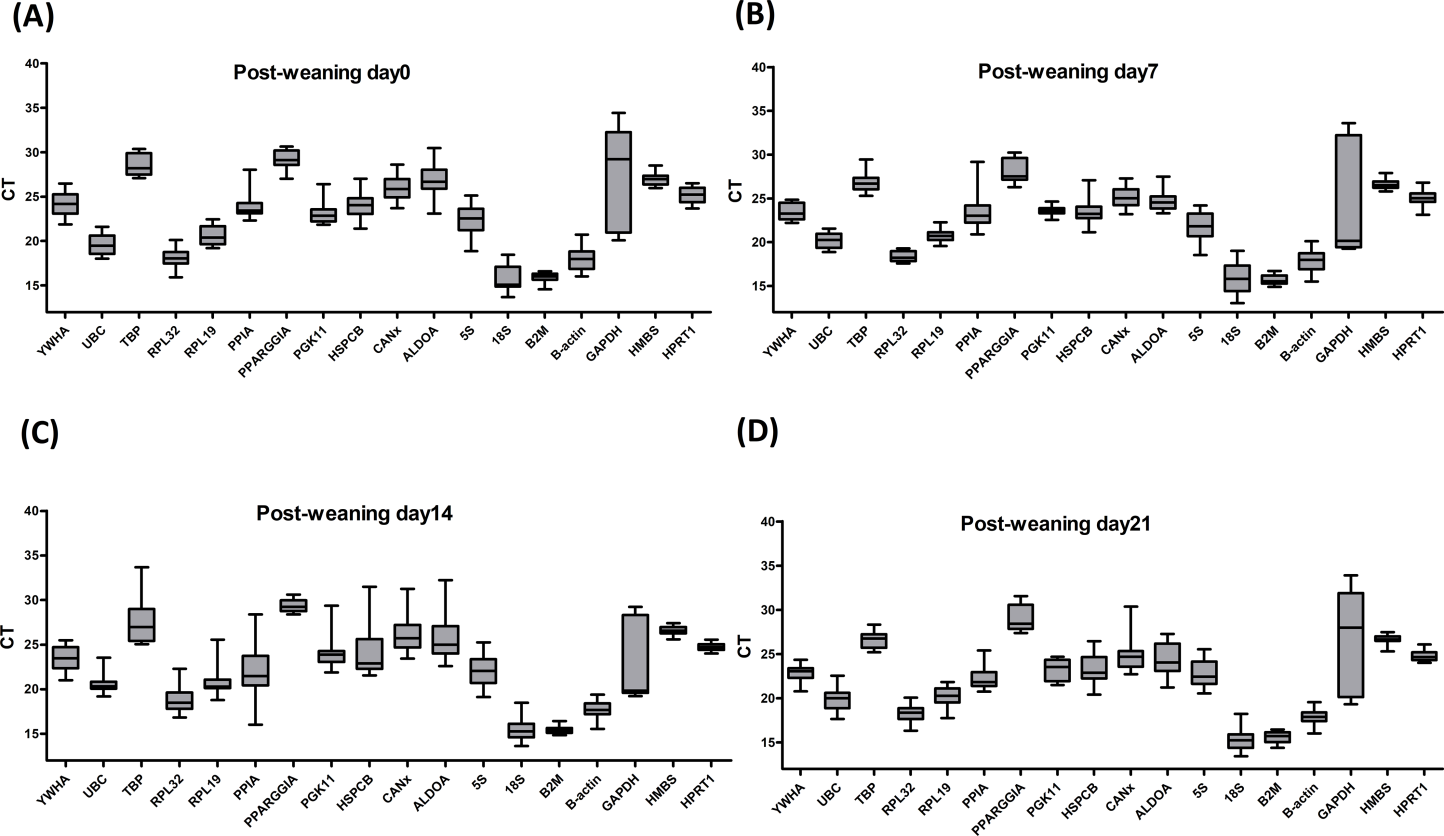
Distribution of the CT values of the reference genes at different growth stages of the gastrointestinal tract. The boxes encompass the 25th to the 75th percentiles. The middle line in a box marks the median. Whisker caps indicate the minimum and maximum values.

The mRNA expression of all the candidate reference genes was assayed in all the mucosa samples from different parts of the GIT. As was shown in Fig 1, the average CT values ranged from 13.70 to 33.90 in the duodenum, 18.48 to 34.25 in the jejunum, 14.19 to 31.55 in the ileum, and 13.62 to 30.25 in the colon. At different growth stages (Fig 2), the average CT values ranged from 13.70 to 34.44 at 0 dpw, 13.05 to 33.60 at 7 dpw, 13.64 to 34.63 at 14 dpw, and 13.44 to 34.01 at 21 dpw. Although the average CT values varied significantly, the overall distribution of the CT values was similar at the four different parts of the GIT. A similar trend in the distribution of the CT values was observed at the different growth stages (0, 7, 14, and 21 dpw).

The CT values of reference genes *HMBS* (*P* >0.05) and *B2M* (*P* >0.05) were not significantly altered at the different parts of the GIT or at the different growth stages, whereas the other 16 reference genes (*P* <0.05) were significantly changed at the different parts of GIT and at different growth stages (Table 3). These results showed that *HMBS* and *B2M* had the most stable CT values compared with the CT values of the other reference genes, implying that both *HMBS* and *B2M* are optimal reference genes.

**Table 3:** A comparison of CT values of the 18 reference genes at different GITs and growth stages.

Gene	GIT				Age (Post-weaning)				SEM			*P*-value		
	Duodenum	Jejunum	Ileum	Colon	Day 0	Day 7	Day 14	Day 21	GIT	Age	GIT*Age	GIT	Age	GIT*Age
*YWHA*	23.61	23.62	23.03	23.61	24.09^a^	23.44^b^	23.47^b^	22.89^c^	0.185	0.185	0.369	0.065	<0.001	<0.001
*UBC*	20.07^b^	20.01^b^	19.69^b^	20.61^a^	19.62^c^	20.26^ab^	20.63^a^	19.86^bc^	0.189	0.189	0.379	0.010	0.002	<0.001
*TBP*	28.09^a^	27.07^b^	27.01^b^	27.49^ab^	28.57^a^	26.84^bc^	27.60^b^	26.65^c^	0.292	0.292	0.585	0.041	<0.001	0.003
*RPL32*	18.73	18.46	18.87	18.05	18.11^b^	18.33^b^	19.29^a^	18.38^b^	0.302	0.302	0.605	0.237	0.037	0.062
*RPL19*	21.39^a^	20.41^b^	19.68^c^	20.91^ab^	20.61^ab^	20.69^ab^	20.93^a^	20.17^b^	0.185	0.185	0.371	<0.001	0.039	<0.001
*PPIA*	23.27	22.76	22.75	22.29	23.73^a^	23.29^a^	21.84^b^	22.20^b^	0.332	0.332	0.664	0.232	<0.001	<0.001
*PPARGGIA*	29.15	28.59	28.77	29.06	29.18^a^	28.15^b^	29.32^a^	28.92^a^	0.207	0.207	0.415	0.209	0.001	<0.001
*PGK11*	24.57^a^	23.48^b^	22.53^c^	23.69^b^	23.19^b^	23.63^ab^	24.21^a^	23.23^b^	0.217	0.217	0.435	<0.001	0.004	0.001
*HSPCB*	25.16^a^	23.06^b^	23.29^b^	23.58^b^	24.12^a^	23.63^ab^	24.11^a^	23.23^b^	0.28	0.28	0.561	<0.001	0.081	<0.001
*CANx*	25.93	25.46	25.3	25.57	25.90a	25.15b	26.28a	24.93b	0.259	0.259	0.518	0.361	0.001	<0.001
*ALDOA*	26.87^a^	24.78^b^	25.09^b^	25.08^b^	26.84^a^	24.75^c^	25.88^b^	24.34^c^	0.29	0.29	0.579	<0.001	<0.001	<0.001
*5S*	22.09^b^	21.12^c^	22.19^b^	23.52^a^	22.41^ab^	21.68^b^	22.01^b^	22.83^a^	0.252	0.252	0.503	<0.001	0.012	<0.001
*18S*	15.72^a^	16.09^a^	15.74^a^	15.07^b^	15.76^ab^	16.03^a^	15.49^ab^	15.35^b^	0.223	0.223	0.446	0.015	0.141	<0.001
*B2M*	15.78	15.67	15.53	15.74	15.92	15.72	15.47	15.61	0.092	0.064	0.103	0.333	0.102	0.081
*B-*^a^*ctin*	17.99^ab^	18.47^a^	17.40^b^	17.77^b^	18.07	17.92	17.71	17.93	0.199	0.199	0.398	0.003	0.654	0.042
*G*^a^*PDH*	24.96^b^	27.23^a^	25.22^b^	23.82^b^	27.33^a^	24.49^b^	22.95^b^	26.46^a^	0.648	0.648	1.296	0.004	<0.001	<0.001
*HMBS*	26.65	26.80	26.61	26.74	26.91	26.64	26.58	26.67	0.099	0.099	0.197	0.508	0.094	0.052
*HPRT1*	25.61^a^	24.73^b^	24.53^b^	24.83^b^	25.22^a^	24.98^ab^	24.71^b^	24.79^b^	0.11	0.11	0.22	<0.001	0.007	0.001

^a,b,c^ Mean values within a row with unlike superscript letters were significantly different (*P*< 0·05).

### GeNorm analysis

**Fig 3:**
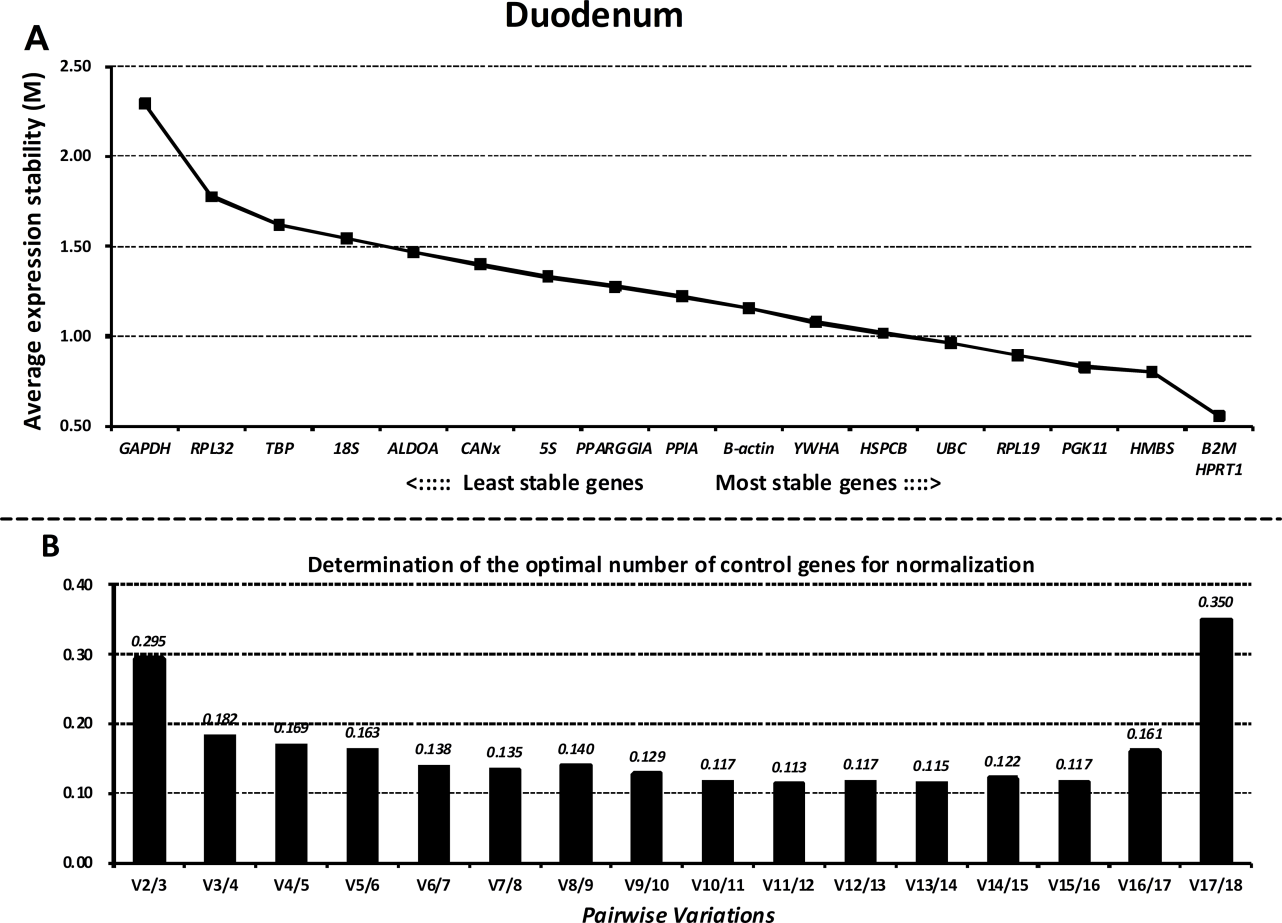
Gene expression stability and ranking of the reference genes in the duodenum as calculated by GeNorm. A) Rank order of gene expression stability based on average expression stability values (M) for the reference genes from least stable (left) to most stable (right). B) Pairwise variation analysis (V) to determine the optimal number of reference genes for RT-qPCR data normalization.

**Fig 4:**
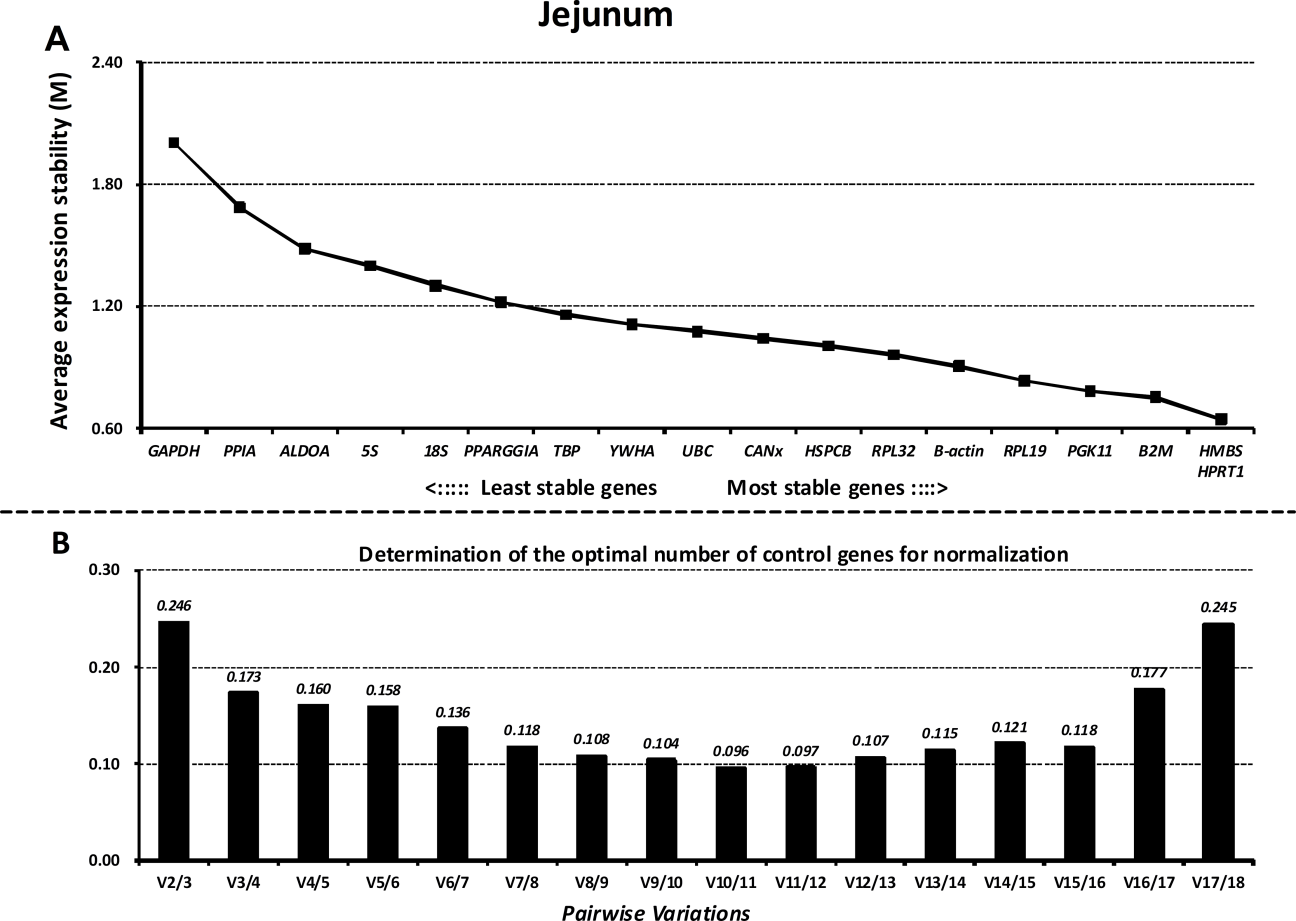
Gene expression stability and rankings of the reference genes in the jejunum as calculated by GeNorm. A) Rank order of gene expression stability based on average expression stability values (M) for the reference genes from least stable (left) to most stable (right). B) Pairwise variation analysis (V) to determine the optimal number of reference genes for RT-qPCR data normalization.

**Fig 5:**
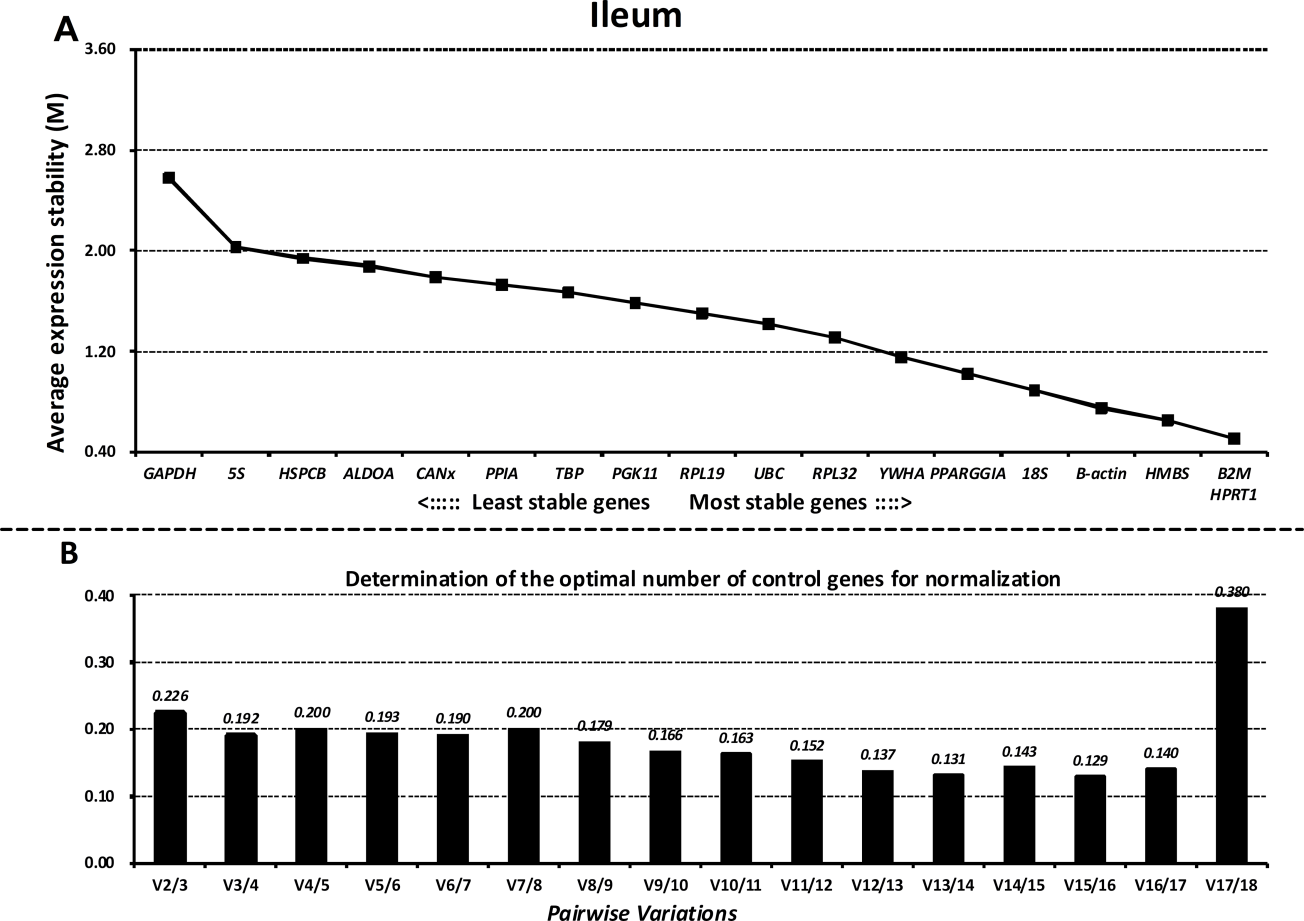
Gene expression stability and rankings of the reference genes in the ileum as calculated by GeNorm. A) Rank order of gene expression stability based on average expression stability values (M) for the reference genes from least stable (left) to most stable (right). B) Pairwise variation analysis (V) to determine the optimal number of reference genes for RT-qPCR data normalization.

**Fig 6:**
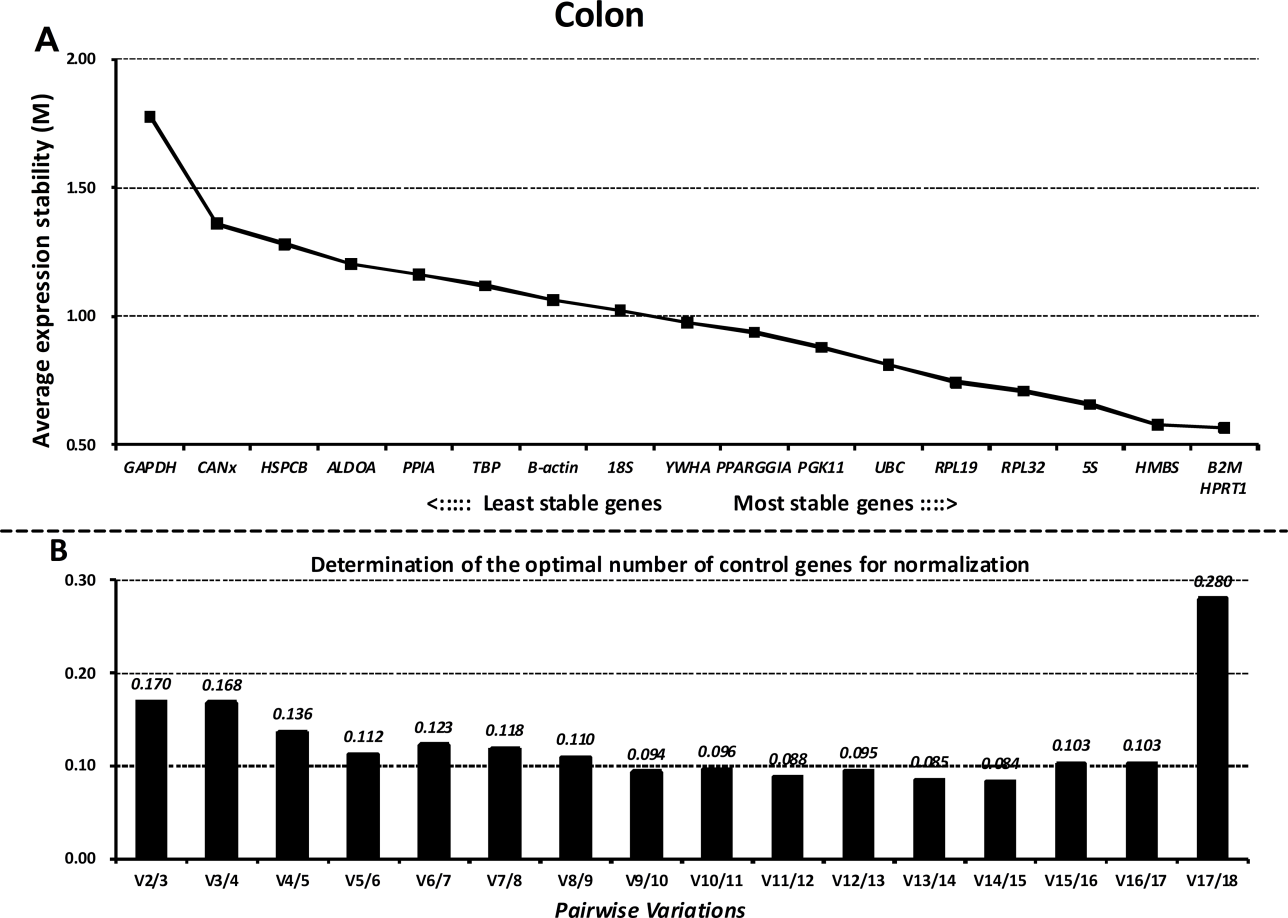
Gene expression stability and rankings of the reference genes in the colon as calculated by GeNorm. A) Rank order of gene expression stability based on average expression stability values (M) for the reference genes from least stable (left) to most stable (right). B) Pairwise variation analysis (V) to determine the optimal number of reference genes for RT-qPCR data normalization.

**Fig 7:**
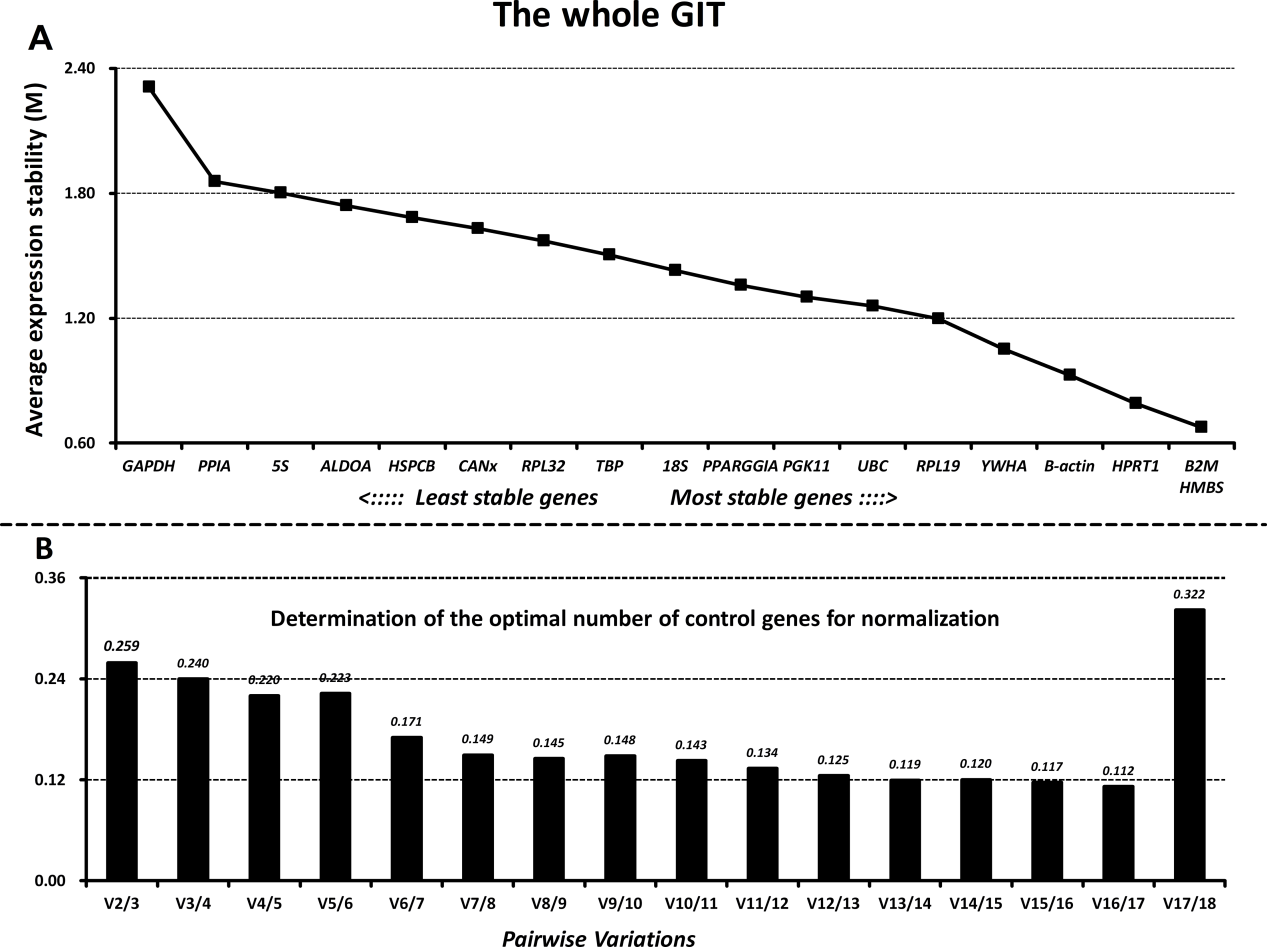
Gene expression stability and rankings of the reference genes in the whole gastrointestinal tract as calculated by GeNorm. A) Rank order of gene expression stability based on average expression stability values (M) for the reference genes from least stable (left) to most stable (right). B) Pairwise variation analysis (V) to determine the optimal number of reference genes for RT-qPCR data normalization.

Next, we ranked all the reference genes based on their stability using GeNorm. The M values of all the reference genes at different parts of the GIT and the whole GIT are shown in Figs 3A–7A. A greater value of M means higher variation in gene expression. At different parts of the GIT as well as the whole GIT, *HMBS*, *B2M*, and *HPRT1* had the lowest M values and the most stable mRNA expression, whereas *GAPDH* exhibited significant variations and was the least stable gene. Moreover, the candidate genes *HPRT1*/*HMBS*/*B2M*, *HMBS*/*RPL32*/*PGK11*, *B2M/ HMBS*/*HPRT1*, and *HPRT1*/*YWHA*/*B2M* had the lowest M values at 0, 7, 14, and 21 dpw, respectively, whereas *GAPDH* again exhibited significant variations and was the least stable gene (S3A–S6A Figs).

Additionally, GeNorm determined the optimal number of reference genes needed to normalize RT-qPCR data on the basis of the average value of pairwise variations (Vn/n+1). In this study, when the cut-off M value was < 0.24, the inclusion of an additional reference gene did not further improve the normalization. On the basis of this threshold, the values of V3/4 values fell below 0.24 for different parts of the GIT (Figs 3B–7B) and different growth stages (S3B–S6B Figs), suggesting that the three selected reference genes (*B2M/HMBS/HPRT*) would be sufficient to normalize the data across all the samples.

### BestKeeper analysis

The correlation of repeated pairwise variations was calculated to assess the optimal reference genes using BestKeeper. BestKeeper requires that the SD values of selected reference genes should not exceed 1.00, and the most stable reference gene also should have a Pearson coefficient of correlation (R) close to 1.00. Table 4 showed the R values of all the reference genes at different parts of the GIT ranked according to the BestKeeper analysis. In the duodenum, *B2M*, *HMBS*, and *PGK11* were the most stable reference genes with R values of 0.97, 0.92, and 0.89 respectively. In the jejunum, *HMBS*, *B2M*, and *HPRT1* were the most stable with R values of 0.87, 0.81, and 0.62 respectively. In the ileum, *B2M*, *HMBS*, and *HSPCB* were the most stable with R values of 0.82, 0.82, and 0.77 respectively. In the colon, *B2M*, *HPRT1*, and *HMBS* were the most stable with R values of 0.79, 0.67, and 0.61 respectively. In the whole GIT, *B2M*, *HMBS*, and *HPRT1* were the most stable with R values of 0.78, 0.70, and 0.59 respectively.

**Table 4:** Reference genes stability calculated by BestKeeper based on the CT.

**a) Duodenum**			**b) Jejunum**		
Gene	Coeff.of corr.[R]	Std dev[± CT]	Gene	Coeff.of corr.[R]	Std dev[± CT]
B2M	0.97	0.56	HMBS	0.87	0.59
HMBS	0.92	0.83	B2M	0.81	0.32
PGK11	0.89	0.81	HPRT1	0.62	0.74
HPRT1	0.87	0.55	RPL19	0.62	0.68
UBC	0.84	0.74	B-actin	0.52	0.99
RPL32	0.59	0.83	PGK11	0.48	0.47
TBP	0.57	0.47	UBC	0.46	0.99
RPL19	0.3	0.93	YWHA	0.1	0.77
B-actin	0.16	0.56			
**c) Ileum**			**d) Colon**		
Gene	Coeff.of corr.[R]	Std dev[± CT]		Gene	Coeff.of corr.[R]	Std dev[± CT]
B2M	0.82	0.52	B2M	0.79	0.52
HMBS	0.82	0.59	HPRT1	0.67	0.47
HSPCB	0.77	0.55	HMBS	0.61	0.64
HPRT1	0.67	0.37	RPL19	0.44	0.62
YWHA	0.53	0.92	PPARGGIA	0.41	0.88
PGK11	0.24	0.61	YWHA	0.36	0.9
RPL19	0.23	0.82	RPL32	0.32	0.59
			5S	0.05	0.98
**e) The whole GIT**					
Gene	coeff. of corr. [r]	std dev [± CP]			
B2M	0.78	0.50			
HMBS	0.70	0.43			
HPRT1	0.59	0.59			
UBC	0.52	0.89			
B-actin	0.43	0.85			
RPL32	0.43	0.94			
YWHA	0.40	0.98			
RPL19	0.17	0.86			

Coeff. of corr. [R]: Pearson coefficient of correlation; Std dev [± CT]: the standard deviation of the CT.

At different growth stages (S2 Table), *PGK11*/*HPRT1*/*B2M* (R values 0.89, 0.79, and 0.76), *HPRT1*/*HMBS*/*TBP* (R values 0.74, 0.68, and 0.66), *HPRT1/PGK11/B2M* (R values 0.95, 0.91, and 0.91), and *B2M/HMBS/HPRT1* (R values 0.66, 0.63, and 0.51) were the most stable reference genes at 0, 7, 14, and 21 dpw, respectively.

### Reference gene validation

**Fig 8:**
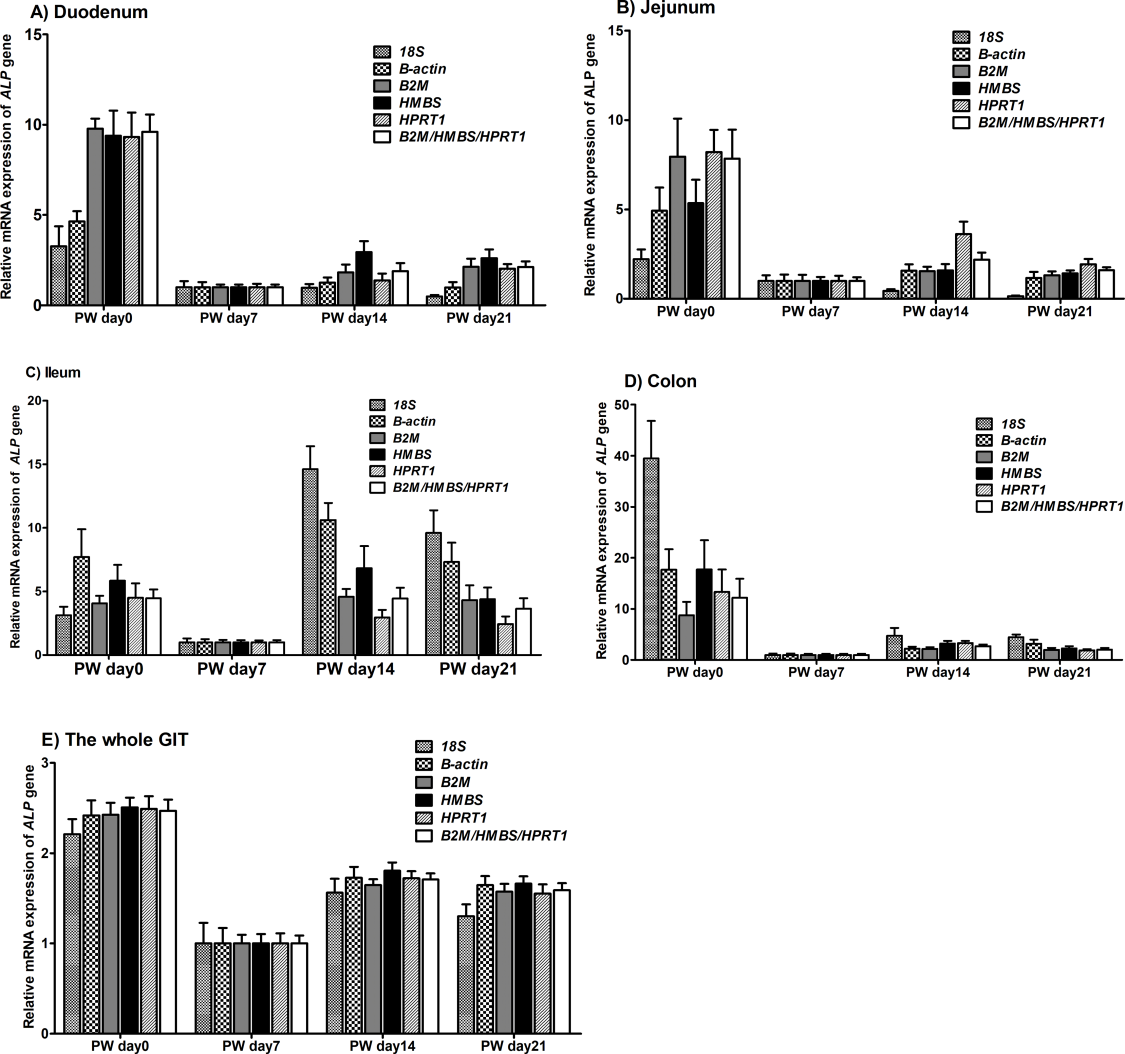
**Effect of normalization on *ALP* expression values in the duodenum (A), jejunum (B), ileum (C), colon (D) and the whole gastrointestinal tract (E) of piglets.** The expression of *ALP* was normalized to 1*8S*, *B-actin*, *HMBS*, *B2M* and *HPRT1* and the geometric mean of *B2M/HMBS/HPRT1* expression and is shown relative to its expression at different post-weaning stages. Error bars indicate mean ± standard error of the mean (SEM).

According to the results of GeNorm and BestKeeper, the optimal number of reference genes in this study are three (*B2M*, *HMBS* and *HPRT1*), and we then used *ALP* as a target gene to assess the reference genes by normalizing its expression against all the reference genes as well as the geometric mean of *B2M/HMBS/HPRT1* (S3–S7 Tables). Generally, the mRNA expression pattern of *ALP* in the duodenum, jejunum, colon and the whole GIT was similar when normalized using *HMBS*, *B2M*, *HPRT1* compared with the geometric mean of *B2M/HMBS/HPRT1* (Fig 8). However, in the ileum, the *ALP* expression obtained by normalized against the geometric mean of *B2M/HMBS/HPRT1* significantly changed when compared with its expression normalized against *HMBS*, *B2M*, *HPRT1* at 0, 14, 21 dpw. Therefore, it suggested that the three selected reference genes (*B2M/HMBS/HPRT*) instead of one individual reference gene (e.g., *HMBS*, *B2M*, *HPRT1*) would be more sufficient to normalize the data across all the samples.

Furthermore, we also assessed the relative mRNA expression of *ALP* normalized against some commonly used reference genes (e.g., *B-actin* and *18S*) and the geometric mean of *B2M/HMBS/HPRT1* (Fig 8). In the duodenum and jejunum, compared with normalization against *B-actin* and *18S*, normalization against the geometric mean of *B2M/HMBS/HPRT1* produced a much higher value (about 2.0-fold) for *ALP* expression at the peak of relative expression at 0 dpw. In the ileum, compared with normalization against *B-actin* and *18S*, the normalization against the geometric mean of *B2M/HMBS/HPRT1*, gave a much lower value for *ALP* expression at 14 and 21 dpw. In the colon, the *ALP* expression obtained by normalized against the geometric mean of *B2M/HMBS/HPRT1* and *B-actin* was significantly lower than its expression normalized against *18S* at 0 dpw. In the whole GIT, the peak of the *ALP* expression normalized against *18S* and *B-actin* did not significantly change when compared with normalization against the geometric mean of *B2M/HMBS/HPRT1*. Thus, at different parts of the GIT, normalization against some commonly used reference genes produced dramatic differences in *ALP* expression that were not seen when normalized against the geometric mean of *B2M/HMBS/HPRT1*.

## Discussion

Because gene expression analysis by RT-qPCR assay has high specificity, accuracy, and is cost-effective, it is commonly used to identify genes associated with novel metabolic or signaling pathways involved in multiple biological processes in pig, which is a good model for human health. It is therefore critical to select appropriate housekeeping reference genes for RT-qPCR data normalization in gene expression analysis [20]. A total of 665 published papers (up to 31 December 2017) involving *Sus scrofa* (i.e., swine, pig, boar, sow, or boar) that were related to mRNA gene expression analysis using miscellaneous technologies were available in NCBI’s PubMed database (S8 Table). In these publications, the three most commonly used housekeeping genes were *B-actin* (158 papers, 23.72%), *GAPDH* (140 papers, 21.02%), and *18S* (67 papers, 10.67%). Less than 25% of the publications used more than one endogenous control gene to assess gene expression, although normalization with a single housekeeping gene can produce false results [21]. Therefore, a set of constant housekeeping genes for pig is important for determining mRNA abundance at different developmental stages, in different tissues, and in environment-specific samples [22]. In this study, we systematically evaluated the reliability of 18 commonly used housekeeping genes across the whole GIT and developed a new set of constant housekeeping genes in pig at the weaning stage. We identified *B2M*, *HMBS*, and *HPRT1* as the optimal housekeeping genes for normalization of RT-qPCR expression data of genes involved in the intestinal development of piglets because of their high expression abundance and high stability in different parts of the GIT (e.g., duodenum, jejunum, ileum, and colon).

*B2M*, is ubiquitously expressed in nucleated cells and is present in most biological fluids, such as urine, synovial fluid, and serum [23,24]. Besides coding the light chain of major histocompatibility complex (MHC) class I molecules, *B2M* also plays multiple roles in the immune system of pigs [24]. In studies that use pig as an animal model, *B2M* was a commonly used reference gene in longissimus thoracis et lumborum muscle [25] and duodenum [7], but it was also identified as the least stable reference gene in other tissues, such as oocytes and preimplantation embryos [26], left ventricle [27], ovary [28], and skeletal muscle [29]. *HPRT1* is conserved across prokaryotes and eukaryotes and plays a vital role in the generation of purine nucleotides via the purine salvage pathway [30]. *HPRT1* is considered to be a somatic cell genetic marker with clinical significance in human disease, and is therefore one of the best characterized genes in human [31]. *HPRT1* is a frequently used housekeeping gene in different pig tissues, such as diaphragm, heart, lung [32], skeletal muscle [8], right and left atrium, left ventricle [27], and duodenum [7]. However, in a study that assessed a set of reference genes for different tissues (liver, lung, kidney, spleen, stomach, small intestine, and large intestine) of different pig breeds (Berkshire, Landrace, Duroc, and Yorkshire), *HPRT1* was the least stable housekeeping gene due to its inconsistent expression patterns [6]. The *HMBS* gene sequence is about 10-kb long, has 15 exons, and encodes a major protein involved in the heme biosynthetic pathway that regulates autosomal dominant disorders, such as acute intermittent porphyria [33]. *HMBS* also stimulates transferase activity that catalyzes the conversion of guanine to guanosine monophosphate and hypoxanthine to inosine monophosphate via the transfer of the 5-phosphoribosyl group from 5-phosphoribosyl 1-pyrophosphate [33]. *HMBS* was identified as the most stable housekeeping gene in the duodenum of piglets [7], but was found to be the least stable housekeeping gene in adipose tissue [22] and uterus [28].

The most frequently used housekeeping genes, *GAPDH*, *B-actin*, and *18S* [9], were not identified as ideal endogenous control genes in this study, agreeing with the findings of Nygard et al. (2007) [34], Cinar et al. (2012) [35], Zhang et al. (2012) [20], Gessner et al. (2013) [36], Li et al. (2016) [37], and Wang et al. (2016) [7], but contradicting the findings of Erkens et al. (2006) [14], Kuijk et al. (2007) [26], and Li et al. (2011) [38]. Because RT-qPCR has dynamic range and increased sensitivity compared with traditional quantitation techniques, the well-known housekeeping genes, especially *GAPDH*, *B-actin*, and *18S*, have been shown to be significantly affected by biological processes, different experimental treatments, and different cell types and tissues [39]. The optimal housekeeping genes identified in this study were different from those chosen in previous studies because the samples that were used varied in cell types [40], tissues [6,32,34], developmental stages [20,29], and breeds [6,15]. Reference genes used as ideal endogenous control genes are therefore those that are commonly assumed to be constant and highly expressed under specific circumstances or in specific tissues [8].

To validate the stability of the housekeeping genes we used GeNorm and BestKeeper. According to GeNorm, *B2M/HMBS/HPRT1* were the optimal housekeeping genes in the duodenum, jejunum, ileum, and colon as well as the whole GIT. According to BestKeeper, *B2M/HMBS/PGK11*, *HMBS/B2M/HPRT1*, *B2M/HMBS/HSPCB*, *B2M/HPRT1/HMBS*, and *B2M/HMBS/HPRT1* had the highest stability in the duodenum, jejunum, ileum, colon and the whole GIT, respectively. In addition to the GeNorm and BestKeeper analyses, we assessed the impact of normalization on the expression pattern of a target gene (*ALP*) using all the reference genes and the geometric mean. *ALP* is an important regulator involved in the primary digestive and absorptive functions of the small intestine and is expressed mainly in the intestinal mucosa of pig [41]. *ALP* is involved in the hydrolysis of monophosphate esters and transcellular solute transport, and participates in the absorption of fat, which is crucial to gut mucosal structures and functions in weaned-piglets [16]. Because of its critical role in intestinal development [7], we selected *ALP* as the target gene to validate the reference genes. Intriguingly, the mRNA expression of *ALP* at different parts of the GIT, normalized against *B2M*, *HMBS*, *HPRT1*, and the geometric mean of all the reference genes, was similar at all post-weaning growth stages tested. However, compared with the results obtained by normalized against *B2M*, *HMBS*, *HPRT1*, and the geometric mean of all the reference genes, underestimation or overestimation of *ALP* expression occurred when the *ALP* RT-qPCR expression data were normalized to other commonly used reference genes, including *GAPDH*, *B-actin*, or *18S*. Thus, substantial differences in the relative expression levels of *ALP* were obtained when different reference genes were chosen for normalization, indicating the need for accurate validation of reference genes.

## Conclusion

The results of the GeNorm and BestKeeper analyses indicated that *B2M/HMBS/HPRT1* were the optimal reference genes in duodenum, jejunum, ileum, and colon, as well as the whole GIT of pig, whereas other commonly used reference genes (e.g., *GAPDH*, *B-actin*, and *18S*) were the least stable. Our results provide a set of optimal endogenous control genes for normalization of RT-qPCR expression data for intestinal studies of pig during the weaning stage.

## Supporting information

S1 Table Sequences of the PCR-amplified DNA fragments obtained using the primers designed for the reference and target genes. (DOCX)

S2 Table Stability of the reference genes at different growth stages of the gastrointestinal tract based on the BestKeeper analysis. (DOCX)

S3 Table Normalization of *ALP* gene expression in the duodenum against the 18 reference genes. (DOCX)

S4 Table Normalization of *ALP* gene expression in the jejunum against the 18 reference genes. (DOCX)

S5 Table Normalization of *ALP* gene expression in the ileum against the 18 reference genes. (DOCX)

S6 Table Normalization of *ALP* gene expression in the colon against the 18 reference genes. (DOCX)

S7 Table Normalization of *ALP* gene expression in the whole gastrointestinal tracts against the 18 reference genes. (DOCX)

S8 Table Housekeeping genes of pig identified in the present and previous studies. (DOCX)

S1 Fig Identification of PCR fragments amplified using gene-specific primers for the reference genes tested. Agarose gel (2%) electrophoresis shows a band at the expected size for each reference gene. M: DNA Marker I (MD101) (100–600 bp), 1: *HMBS*, 2: *ALP*, 3: *18S*, 4: *TBP*, 5: *RPL19*, 6: *PPARGC1A*, 7: *5S*, 8: *CANX*, 9: *HSPCB*, 10: *B-actin*, 11: *UBC*, 12: *B2M*, 13: *HPRT1*, 14: *PGK1*, 15: *YWHAZ*, 16: *GAPDH*, 17: *PPIA*, 18: *RPL32*, and 19: *ALDOA*.
(DOCX)

S2 Fig Melting and standard curves of the 18 reference genes and *ALP* target gene. (DOCX)

S3 Fig Gene expression stability and rankings of the reference genes at 0 days post-weaning as calculated by GeNorm. A) Rank order of gene expression stability based on average expression stability values (M) for the reference genes from least stable (left) to most stable (right). B) Pairwise variation analysis (V) to determine the optimal number of reference genes for RT-qPCR data normalization.
(DOCX)

S4 Fig Gene expression stability and rankings of reference genes at 7 days post-weaning as calculated by GeNorm. A) Rank order of gene expression stability based on average expression stability values (M) for the reference genes from least stable (left) to most stable (right). B) Pairwise variation analysis (V) to determine the optimal number of reference genes for RT-qPCR data normalization.
(DOCX)

S5 Fig Gene expression stability and rankings of reference genes at 14 days post-weaning as calculated by GeNorm. A) Rank order of gene expression stability based on average expression stability values (M) for the reference genes from least stable (left) to most stable (right). B) Pairwise variation analysis (V) to determine the optimal number of reference genes for RT-qPCR data normalization.
(DOCX)

S6 Fig Gene expression stability and rankings of reference genes at 21 days post-weaning as calculated by GeNorm. A) Rank order of gene expression stability based on average expression stability values (M) for the reference genes from least stable (left) to most stable (right). B) Pairwise variation analysis (V) to determine the optimal number of reference genes for RT-qPCR data normalization.
(DOCX)
