# Supplementary material for: A new set of reference housekeeping genes for the normalization RT-qPCR data from the intestine of piglets during weaning
Source: PLoS One. 2018 Sep 26;13(9):e0204583. doi: 10.1371/journal.pone.0204583 (PMC6157878; doi:10.1371/journal.pone.0204583)
Supplement: S1 Table — (DOCX) [file pone.0204583.s001.docx]

**S1 Table. Sequencing results of PCR products from the amplification of primers of reference and target genes designed for this experiment.**

| Genes | Sequence |
| --- | --- |
| *YWHA* | GGGGGGCTGACTTACTTCTCTGTGTTCTATTATGAGATTCTGAACTCCCCAGAGAAAGCC  TGCTCTCTTGCAAAGACAGCATTTGATGAAGCCATTGCTGAACTTGATACATTAAGTGAA  GAGTCATACAAAGACAGCACGCTAATAATGCA |
| *UBC* | TGGAGCCCAG CGACACCATC TAGAACGTCA AGGCGAAGAT CCAAGACAAG GAAGG |
| *TBP* | TTGGTGAGCTCTGCCGCGACCCACAGGGGTGCCGCAGTTTCTGCACAGAGAACTCCAGGAG  CGTTCCTGTAAGTTGCTCGTACTGTGCTGCTACAACG |
| *RPL32* | GAGATCTCCGGCACAGTCAGACCGATATGCAAATTAAGCGGAACTGGCGGAAACCCAGAG  GCATTGACAACAGGGTGCGGAGAAGGTCAAAGGCCAGATCTTGATGCCCAACATTGGTTA  TGGGAGCAACAA |
| *RPL19* | CGAAAGGGCA GGCATATGGG GATCGGTAAG CGGAAGGGTA CTGCCAATGC |
| *PPIA* | TGATGCTTAGATATTCCAGGATTTATGTGCCAGGGTGGTGACTTCACACGCCATAATGGCA  CTGGTGGCAAGTCCATCTATGGAGAGAAATTTGATGATGAGAATTTTATCCTGAAGCATA  CGGGTCCTGGCATCTTGTCCAT |
| *PPARGCIA* | ACCCGTTTGAGAGTGACGCTTGACGAGCGCTTCAGGAGCTGGATGGCGTGGGACATGTGCA  ACCAGGACTCTGAGA |
| *PGK11* | TTCTGTCGCGATTGATGGGAGTTTTGCCCAGGACCAAAGCCCTCATGGATGAGTGTGAAAG  CCACTTCCAGGGGCTGCATCACCATCATAGGTGGTGGAGACACTGCTACCTGCTGTGCCAA  ATGGAACACGGAGGATAAAGTCAA |
| *HSPCB* | GGCTCTCGAGGCTTCTCAAATCTAAAGCTTGGATCCATGAGGACTCCACTAATCGGCGGCG  CCTTTCTGAGCTGCTGCGCTACCATACCTCCCAGTCTGA |
| *CANX* | CCTCCTCGCTGGGGCTGCTGAGCAGGTGTGTGGGGAGTGTTGAGGCAGCCGAGGAGCGCCC  ATGGCTCTGGGTGGTTTATATTTTGACTGTGGCATTACCTGTGTTA |
| *ALDOA* | GTACCTCTGAGGCTGTAGAGCGCTGTGCCAGTACAGAAGGACGGAGCTGACTTTGCCAAGT  GGCGCTGTGTGCTGAAGATCGGGGAGCACACCCCCTCCTCCCTCGCCATCAT |
| *5S* | GTGCAGTCGGGCTGGTTAGTACTTGGATGGGAGACCGCCTGGGAATACCGGGT  GCTGTAGGCTC |
| *18S* | ATTTATATCTGTCGTGTCCGGGCCGGGTGAGGTTTCCCGTGTTGAGTCAAATTAAGCCGCA  GGCTCCACTCCTGGTGGTGCCCTTCCGTCAAA |
| *B2M* | GCGATCGGGCTCCAGAGATTATGAGATGCTGCATCTGGGTTGGATGAATCCAAATTCT  GATTTGTTGCTTTTTAATACTGATAAGCTTTTATACTTTATGCACATAAATCAGAAATC  GTATTGATGTTACCAAAC |
| *B-actin* | GGGGTGCGAGATGAGATCAGATCATCGCGCCTCCAGAGCGCAAGTACTCCGTGTGGATCG  GCGGCTCCATCCTGGCCTCGCTGTCCACCTTCCAGCAGATA |
| *GAPDH* | GCCAACATCA AATGGGGCGA ACTGGTGCT ACGTATGTTG TGGAGTCCAC TGGTGTCCT |
| *HMBS* | GCGGAACTGAATTGAATGGTGCAGAGAGCATGCAAGAGACTATGCAGCGCCACTCATCAG |
| *HPRT1* | TATTTCGGTAGCAATACAAAGCCTAAGAGGAGAGTTCAAGTTGAGTTTGGAAACATCTGA |
| *ALP* | GAAAAGCAGAAGCCCTGGCATGGACCGATTCCCGTACCTGGCTCTGTCCAAGACATACAA  CGTGGACAGACAGGTGA |
