# Supplementary material for: A new set of reference housekeeping genes for the normalization RT-qPCR data from the intestine of piglets during weaning
Source: PLoS One. 2018 Sep 26;13(9):e0204583. doi: 10.1371/journal.pone.0204583 (PMC6157878; doi:10.1371/journal.pone.0204583)
Supplement: S2 Table — (DOCX) [file pone.0204583.s002.docx]

**S2 Table. Stability of the reference genes at different growth stages based on the BestKeeper analysis.**

| 1. PW day0 | | |  | 1. PW day7 | | |
| --- | --- | --- | --- | --- | --- | --- |
| Gene | Coeff.of corr.[R] | Std dev[±CT] |  | Gene | Coeff.of corr.[R] | Std dev[± CT] |
| PGK11 | 0.89 | 0.61 |  | HPRT1 | 0.74 | 0.89 |
| HPRT1 | 0.79 | 0.74 |  | HMBS | 0.68 | 0.39 |
| B2M | 0.76 | 0.57 |  | TBP | 0.66 | 0.88 |
| PPIA | 0.75 | 0.79 |  | B2M | 0.62 | 0.55 |
| HMBS | 0.70 | 0.64 |  | RPL19 | 0.58 | 0.56 |
| RPL19 | 0.66 | 0.80 |  | YWHA | 0.54 | 0.81 |
| UBC | 0.66 | 0.88 |  | PGK11 | 0.11 | 0.53 |
| GAPDH | 0.62 | 4.38 |  | RPL32 | 0.05 | 0.56 |
| RPL32 | 0.53 | 0.91 |  |  |  |  |
| 1. PW day14 | | |  | 1. **PW day21** | | |
| Gene | Coeff.of corr.[R] | Std dev[± CT] |  | Gene | Coeff.of corr.[R] | Std dev[± CT] |
| HPRT1 | 0.95 | 0.44 |  | B2M | 0.66 | 0.66 |
| PGK11 | 0.95 | 0.69 |  | HMBS | 0.63 | 0.49 |
| B2M | 0.91 | 0.46 |  | HPRT1 | 0.51 | 0.52 |
| PPARGGIA | 0.58 | 0.57 |  | YWHA | 0.49 | 0.72 |
| UBC | 0.37 | 0.42 |  | TBP | 0.28 | 0.89 |
| HMBS | 0.35 | 0.45 |  |  |  |  |
| B-actin | 0.31 | 0.83 |  |  |  |  |
| RPL19 | 0.22 | 0.61 |  |  |  |  |
