# Supplementary material for: A new set of reference housekeeping genes for the normalization RT-qPCR data from the intestine of piglets during weaning
Source: PLoS One. 2018 Sep 26;13(9):e0204583. doi: 10.1371/journal.pone.0204583 (PMC6157878; doi:10.1371/journal.pone.0204583)
Supplement: S3 Table — (DOCX) [file pone.0204583.s003.docx]

**S3 Table. Normalization of *ALP* gene expression in the duodenum against the 18 reference genes.**

|  | | | | | |  |
| --- | --- | --- | --- | --- | --- | --- |
|  | Age (post-weaning) | | | |  |  |
| Gene | Day 0 | Day 7 | Day 14 | Day 21 | SEM | *P*-value |
| *YWHA* | 26.95^a^ | 1.00^b^ | 1.29^b^ | 1.18^b^ | 3.843 | <0.001 |
| *UBC* | 15.15^a^ | 1.00^b^ | 17.57^a^ | 2.06^b^ | 1.751 | 0.017 |
| *TBP* | 47.71^a^ | 1.00^b^ | 139.36^c^ | 3.70^b^ | 1.299 | 0.046 |
| *RPL32* | 6.49^a^ | 1.00^b^ | 13.80^c^ | 1.28^b^ | 2.150 | 0.008 |
| *RPL19* | 15.58^a^ | 1.00^b^ | 45.50^c^ | 3.25^b^ | 1.790 | 0.016 |
| *PPIA* | 83.81 | 1.00 | 52.18 | 2.08 | 0.595 | 0.256 |
| *PPARGGIA* | 9.16^a^ | 1.00^b^ | 3.72^bc^ | 5.90^c^ | 2.153 | 0.008 |
| *PGK11* | 26.06^a^ | 1.00^b^ | 70.93^c^ | 2.76^a^ | 1.546 | 0.027 |
| *HSPCB* | 88.14 | 1.00 | 365.00 | 16.05 | 0.852 | 0.134 |
| *CANx* | 64.47^a^ | 1.00^b^ | 120.63^c^ | 2.07^b^ | 2.078 | 0.009 |
| *ALDOA* | 338.76^a^ | 1.00^b^ | 270.35^a^ | 8.06^b^ | 1.731 | 0.018 |
| *5S* | 17.69^a^ | 1.00^b^ | 1.22^b^ | 16.99^a^ | 2.017 | 0.010 |
| *18S* | 3.26^a^ | 1.00^b^ | 0.97^b^ | 0.50^b^ | 1.813 | 0.015 |
| *B2M* | 9.78^a^ | 1.00^b^ | 1.82^b^ | 2.13^b^ | 1.407 | <0.001 |
| *B-actin* | 4.65^a^ | 1.00^b^ | 1.26^b^ | 0.99^b^ | 1.393 | <0.001 |
| *GAPDH* | 86.65^a^ | 1.00^b^ | 2.38^b^ | 76.89^a^ | 1.555 | 0.026 |
| *HMBS* | 9.40^a^ | 1.00^b^ | 2.94^b^ | 2.61^b^ | 1.845 | <0.001 |
| *HPRT1* | 9.33^a^ | 1.00^b^ | 1.38^b^ | 2.03^b^ | 2.438 | <0.001 |
| *Geomean*^1^ | 9.60^a^ | 1.00^b^ | 1.89^b^ | 2.12^b^ | 0.791 | <0.001 |

**Note:** ^a,b,c^ Means within the same row without common superscripts differ significantly (*P* < 0.05) .

^1^ Means the geomean of *B2M*/*HMBS*/*HPRT1*.
