# Supplementary material for: A new set of reference housekeeping genes for the normalization RT-qPCR data from the intestine of piglets during weaning
Source: PLoS One. 2018 Sep 26;13(9):e0204583. doi: 10.1371/journal.pone.0204583 (PMC6157878; doi:10.1371/journal.pone.0204583)
Supplement: S4 Table — (DOCX) [file pone.0204583.s004.docx]

**S4 Table. Normalization of *ALP* gene expression in the jejunum against the 18 reference genes.**

|  | Age (post-weaning) | | | |  |  |
| --- | --- | --- | --- | --- | --- | --- |
| Gene | Day 0 | Day 7 | Day 14 | Day 21 | SEM | *P*-value |
| *YWHA* | 6.70^a^ | 1.00^b^ | 9.99^a^ | 2.15^b^ | 0.622 | 0.001 |
| *UBC* | 2.65^a^ | 1.00^b^ | 1.95^ab^ | 1.82^ab^ | 1.255 | 0.050 |
| *TBP* | 19.53^a^ | 1.00^b^ | 9.44^c^ | 1.03^b^ | 1.275 | <0.001 |
| *RPL32* | 2.98^a^ | 1.00^b^ | 2.97^a^ | 2.75^a^ | 1.049 | 0.043 |
| *RPL19* | 3.44^a^ | 1.00^b^ | 1.79^b^ | 1.59^b^ | 0.351 | 0.013 |
| *PPIA* | 11.06^a^ | 1.00^b^ | 6.23^c^ | 1.35^b^ | 2.285 | 0.006 |
| *PPARGGIA* | 14.59^a^ | 1.00^b^ | 9.24^c^ | 4.14^d^ | 0.860 | 0.032 |
| *PGK11* | 2.60^a^ | 1.00^b^ | 2.02^ab^ | 1.18^b^ | 0.655 | 0.003 |
| *HSPCB* | 2.92^a^ | 1.00^b^ | 1.14^b^ | 2.30^ab^ | 0.779 | 0.002 |
| *CANx* | 7.45 | 1.00 | 2.66 | 19.81 | 0.775 | 0.162 |
| *ALDOA* | 33.36^a^ | 1.00^b^ | 5.16^c^ | 6.06^c^ | 1.768 | 0.017 |
| *5S* | 3.31^a^ | 1.00^b^ | 4.07^a^ | 2.96^ab^ | 1.149 | 0.065 |
| *18S* | 2.21^a^ | 1.00^ab^ | 0.44^b^ | 0.14^b^ | 1.053 | <0.001 |
| *B2M* | 7.95^a^ | 1.00^b^ | 1.54^b^ | 1.31^b^ | 1.607 | <0.001 |
| *B-actin* | 4.92^a^ | 1.00^b^ | 1.57^b^ | 1.16^b^ | 3.390 | <0.001 |
| *GAPDH* | 8.17^a^ | 1.00^b^ | 0.01^b^ | 0.20^b^ | 18.852 | <0.001 |
| *HMBS* | 5.34^a^ | 1.00^b^ | 1.58^b^ | 1.42^b^ | 0.654 | <0.001 |
| *HPRT1* | 8.21^a^ | 1.00^b^ | 3.61^c^ | 1.92^bc^ | 1.234 | <0.001 |
| *Geomean*^1^ | 7.84^a^ | 1.00^b^ | 2.19^b^ | 1.60^b^ | 0.753 | <0.001 |

**Note:** ^a,b,c^ Means within the same row without common superscripts differ significantly (*P* < 0.05) .

^1^ Means the geomean of *B2M*/*HMBS*/*HPRT1*.
