# Supplementary material for: A new set of reference housekeeping genes for the normalization RT-qPCR data from the intestine of piglets during weaning
Source: PLoS One. 2018 Sep 26;13(9):e0204583. doi: 10.1371/journal.pone.0204583 (PMC6157878; doi:10.1371/journal.pone.0204583)
Supplement: S5 Table — (DOCX) [file pone.0204583.s005.docx]

**S5 Table. Normalization of *ALP* gene expression in the ileum against the 18 reference genes.**

|  | | | | | |  |
| --- | --- | --- | --- | --- | --- | --- |
|  | Age (post-weaning) | | | |  |  |
| Gene | Day 0 | Day 7 | Day 14 | Day 21 | SEM | *P*-value |
| *YWHA* | 12.13^a^ | 1.00^b^ | 4.12^c^ | 4.70^c^ | 2.062 | 0.009 |
| *UBC* | 1.76^ab^ | 1.00^b^ | 2.21^a^ | 1.01^b^ | 1.532 | 0.027 |
| *TBP* | 37.33^a^ | 1.00^b^ | 7.56^c^ | 9.27^c^ | 1.333 | 0.043 |
| *RPL32* | 12.90^a^ | 1.00^b^ | 13.86^a^ | 11.39^a^ | 0.910 | 0.016 |
| *RPL19* | 3.94^a^ | 1.00^b^ | 2.55^ab^ | 1.12^b^ | 1.355 | <0.001 |
| *PPIA* | 0.67 | 1.00 | 0.06 | 0.49 | 0.407 | 0.415 |
| *PPARGGIA* | 24.20^ac^ | 1.00^b^ | 10.49^c^ | 37.35^a^ | 1.212 | 0.026 |
| *PGK11* | 3.44^a^ | 1.00^b^ | 2.44^ab^ | 1.32^b^ | 0.981 | 0.011 |
| *HSPCB* | 11.82^a^ | 1.00^b^ | 2.26^b^ | 3.38^b^ | 3.129 | <0.001 |
| *CANx* | 8.39^a^ | 1.00^b^ | 1.74^b^ | 3.53^b^ | 2.456 | <0.001 |
| *ALDOA* | 16.16^a^ | 1.00^b^ | 0.61^b^ | 0.51^b^ | 2.087 | 0.009 |
| *5S* | 51.58^a^ | 1.00^b^ | 38.48^a^ | 7.13^c^ | 1.347 | 0.041 |
| *18S* | 3.11^a^ | 1.00^b^ | 14.61^c^ | 9.59^d^ | 0.901 | 0.119 |
| *B2M* | 4.07^a^ | 1.00^b^ | 4.58^a^ | 4.31^a^ | 1.916 | 0.012 |
| *B-actin* | 7.69^a^ | 1.00^b^ | 10.60^c^ | 7.31^a^ | 0.904 | 0.018 |
| *GAPDH* | 5394.17^a^ | 1.00^b^ | 1490.24^c^ | 1376.18^c^ | 2.098 | 0.008 |
| *HMBS* | 5.83^a^ | 1.00^b^ | 6.81^a^ | 4.39^a^ | 1.015 | 0.011 |
| *HPRT1* | 4.50^a^ | 1.00^b^ | 2.95^c^ | 2.43^c^ | 0.844 | 0.007 |
| *Geomean*^1^ | 4.45^a^ | 1.00^b^ | 4.51^a^ | 3.63^a^ | 0.597 | 0.006 |

**Note:** ^a,b,c^ Means within the same row without common superscripts differ significantly (*P* < 0.05) .

^1^ Means the geomean of *B2M*/*HMBS*/*HPRT1*.
