# Supplementary material for: A new set of reference housekeeping genes for the normalization RT-qPCR data from the intestine of piglets during weaning
Source: PLoS One. 2018 Sep 26;13(9):e0204583. doi: 10.1371/journal.pone.0204583 (PMC6157878; doi:10.1371/journal.pone.0204583)
Supplement: S6 Table — (DOCX) [file pone.0204583.s006.docx]

**S6 Table. Normalization of *ALP* gene expression in the colon against the 18 reference genes.**

|  | | | | | |  |
| --- | --- | --- | --- | --- | --- | --- |
|  | Age (post-weaning) | | | |  |  |
| Gene | Day 0 | Day 7 | Day 14 | Day 21 | SEM | *P*-value |
| *YWHA* | 7.06^a^ | 1.00^b^ | 2.99^b^ | 0.87^b^ | 1.499 | 0.007 |
| *UBC* | 11.00^a^ | 1.00^b^ | 4.03^c^ | 3.72^c^ | 1.863 | 0.014 |
| *TBP* | 12.39^a^ | 1.00^b^ | 0.96^b^ | 0.55^b^ | 1.146 | 0.046 |
| *RPL32* | 5.29^a^ | 1.00^b^ | 2.77^b^ | 2.19^b^ | 1.576 | 0.007 |
| *RPL19* | 7.45^a^ | 1.00^b^ | 1.56^b^ | 1.30^b^ | 11.666 | <0.001 |
| *PPIA* | 11.60^a^ | 1.00^b^ | 0.87^b^ | 1.17^b^ | 2.187 | 0.007 |
| *PPARGGIA* | 8.05^a^ | 1.00^b^ | 2.97^b^ | 0.80^b^ | 1.451 | 0.033 |
| *PGK11* | 8.35^a^ | 1.00^b^ | 4.56^ab^ | 3.23^ab^ | 1.073 | 0.078 |
| *HSPCB* | 1.12^a^ | 1.00^a^ | 0.47^ab^ | 0.17^b^ | 0.618 | 0.023 |
| *CANx* | 3.51^a^ | 1.00^ab^ | 10.62^c^ | 0.33^b^ | 0.572 | 0.047 |
| *ALDOA* | 8.80^a^ | 1.00^b^ | 3.41^c^ | 1.23^b^ | 2.706 | <0.001 |
| *5S* | 5.25^a^ | 1.00^b^ | 2.48^b^ | 2.16^b^ | 1.375 | 0.039 |
| *18S* | 39.49^a^ | 1.00^b^ | 4.76^c^ | 4.64^c^ | 1.806 | 0.015 |
| *B2M* | 8.76^a^ | 1.00^b^ | 2.17^c^ | 1.99^bc^ | 2.867 | 0.002 |
| *B-actin* | 17.67^a^ | 1.00^b^ | 2.21^bc^ | 3.17^c^ | 1.657 | 0.021 |
| *GAPDH* | 0.01^a^ | 1.00^b^ | 0.20^a^ | 0.01^a^ | 1.413 | 0.036 |
| *HMBS* | 17.72^a^ | 1.00^b^ | 3.22^b^ | 2.28^b^ | 3.018 | 0.002 |
| *HPRT1* | 13.36^a^ | 1.00^b^ | 3.32^c^ | 1.87^b^ | 2.759 | 0.002 |
| *Geomean*^1^ | 12.19^a^ | 1.00^b^ | 2.70^b^ | 2.06^b^ | 1.022 | 0.001 |

**Note:** ^a,b,c^ Means within the same row without common superscripts differ significantly (*P* < 0.05) .

^1^ Means the geomean of *B2M*/*HMBS*/*HPRT1*.
