# Supplementary material for: A new set of reference housekeeping genes for the normalization RT-qPCR data from the intestine of piglets during weaning
Source: PLoS One. 2018 Sep 26;13(9):e0204583. doi: 10.1371/journal.pone.0204583 (PMC6157878; doi:10.1371/journal.pone.0204583)
Supplement: S7 Table — (DOCX) [file pone.0204583.s007.docx]

**S7 Table. Normalization of ALP gene expression in the whole gastrointestinal tract against the 18 reference genes.**

|  | Age (post-weaning) | | | |  |  |
| --- | --- | --- | --- | --- | --- | --- |
| Gene | Day 0 | Day 7 | Day 14 | Day 21 | SEM | *P*-value |
| *YWHA* | 2.67^a^ | 1.00^b^ | 1.85^ab^ | 1.37^b^ | 1.496 | <0.001 |
| *UBC* | 2.03^a^ | 1.00^b^ | 2.02^a^ | 1.45^ab^ | 2.278 | <0.001 |
| *TBP* | 3.05^a^ | 1.00^b^ | 2.21^a^ | 1.55^ab^ | 1.253 | 0.001 |
| *RPL32* | 2.24^a^ | 1.00^b^ | 2.11^a^ | 1.68^ab^ | 2.195 | <0.001 |
| *RPL19* | 2.31^a^ | 1.00^b^ | 1.95^ab^ | 1.38^ab^ | 2.451 | <0.001 |
| *PPIA* | 2.57^a^ | 1.00^b^ | 1.11^b^ | 1.10^b^ | 0.568 | 0.005 |
| *PPARGGIA* | 2.11^a^ | 1.00^b^ | 2.23^a^ | 2.03^a^ | 3.437 | <0.001 |
| *PGK11* | 2.12^a^ | 1.00^b^ | 2.12^a^ | 1.45^ab^ | 4.399 | <0.001 |
| *HSPCB* | 2.59^a^ | 1.00^b^ | 2.07^a^ | 1.44^ab^ | 1.106 | 0.002 |
| *CANx* | 2.72^a^ | 1.00^b^ | 2.39^a^ | 1.53^ab^ | 2.147 | <0.001 |
| *ALDOA* | 3.40^a^ | 1.00^b^ | 2.40^a^ | 1.45^ab^ | 0.498 | 0.048 |
| *5S* | 2.71^a^ | 1.00^b^ | 2.00^ab^ | 2.22^a^ | 1.041 | 0.003 |
| *18S* | 2.21^a^ | 1.00^b^ | 1.56^ab^ | 1.30^ab^ | 1.873 | <0.001 |
| *B2M* | 2.43^a^ | 1.00^b^ | 1.65^ab^ | 1.57^ab^ | 0.425 | <0.001 |
| *B-actin* | 2.42^a^ | 1.00^b^ | 1.73^ab^ | 1.65^ab^ | 1.368 | <0.001 |
| *GAPDH* | 3.29 | 1.00 | 1.06 | 1.55 | 0.257 | 0.294 |
| *HMBS* | 2.51^a^ | 1.00^b^ | 1.81^ab^ | 1.66^ab^ | 1.142 | <0.001 |
| *HPRT1* | 2.49^a^ | 1.00^b^ | 1.72^ab^ | 1.55^ab^ | 1.666 | <0.001 |
| *Geomean*^1^ | 2.47^a^ | 1.00^b^ | 1.71^c^ | 1.59^c^ | 0.169 | <0.001 |

**Note:** ^a,b,c^ Means within the same row without common superscripts differ significantly (*P* < 0.05) .

^1^ Means the geomean of *B2M*/*HMBS*/*HPRT1*.
