# Supplementary material for: A new set of reference housekeeping genes for the normalization RT-qPCR data from the intestine of piglets during weaning
Source: PLoS One. 2018 Sep 26;13(9):e0204583. doi: 10.1371/journal.pone.0204583 (PMC6157878; doi:10.1371/journal.pone.0204583)
Supplement: S8 Table — (DOCX) [file pone.0204583.s008.docx]

**S8 Table. The housekeeping genes of pig identified in the present and previous studies.**

| References | Selected genes | Most stable genes | Most unstable genes | Sample category |
| --- | --- | --- | --- | --- |
| *Erkens et al. (2006)[1]* | *B-actin,B2M,GAPDH,HMBS,HPRT1,RPL13A,SDHA,TBP,TOP2B* and *YWHAZ* | *B-actin/TBP/TOP2B* | *RPL13A /SDHA* | Backfat and l*ongissimus dorsi* muscle |
| *Kuijk et al. (2007)[2]* | *B2M,B-actin,GAPDH,H2A,PGK1,18S* and *UBC* | *GAPDH/UBC* | *B2M/B-actin/H2A* | Oocytes and preimplantation embryos |
| *Nygard et al. (2007)[3]* | *B-actin,GAPDH,HMBS,HPRT1,RPL4,SDHA,TBP* and *YWHAZ* | *B-actin /RPL4/TBP* | *GAPDH* | Adipose, bladder, bone marrow, cerebellum, cortex cerebri, heart, hippocampus, kidney, liver, lung, lymph nodes, muscle, pancreas, skin, small intestine, stomach and thymus |
| *Svobodova et al.(2008)[4]* | *EEF1A1,GAPDH,HPRT1* and *TOP2B* | *EEF1A1/HPRT1* | *GAPDH* | Diaphragm |
|  |  | *GAPDH/HPRT1* | *EEF1A1* | Heart |
|  |  | *EEF1A1/TOP2B* | *GAPDH* | Kidney |
|  |  | *GAPDH/TOP2B* | *EEF1A1* | Liver |
|  |  | *HPRT1/TOP2B* | *GAPDH* | Lungs |
|  |  | *GAPDH* | *EEF1A1/HPRT1* | Muscle and spleen |
| *Tramontana et al. (2008)[5]* | *VAPB,TRIM41,SCD,RPS15A,MTG1,MRPL39,LTF,FABP3,CSN1S2, API5,AP1S1* and *ACTB* | *MRPL39/API5/VAPB* | *AP1S1* | Mammary tissue |
| *Feng et al.(2010)[6]* | *GPX1,PPIA,GAPDH,SDHA,eEF-1γ,ATP5G1,SHAS2,GPI, B-actin* and *HPRT1* | *PPIA/HPRT1/SDHA* | *SHAS2/GAPDH* | Skeletal muscle of Yorkshire sows |
|  |  | *PPIA/eEF-1γ/HPRT1* | *SHAS2/GAPDH* | Skeletal muscle in Meishan sows |
| *McBryan et al.(2010)[7]* | *B-actin,ATP5G1,B2M,GPX1,RPL4,TBP and YWHAZ* | *B2M/RPL4/TBP* | *GPX1/ B-actin* | *Longissimus thoracis et lumborum* muscle |
| *Gu et al.(2011)[8]* | *5S,18S, B-actin,ALDOA,B2M,GAPDH,HMBS,HPRT1,*  *HSPCB,Met-tRNA,PGK1,PPIA,RPL13A,RPL19,*  *SDHA,TBP,TOP2B,U6,UBC* and *YWHAZHMBS* | *HSPCB/ALDOA/GAPDH*  *TOP2B/HSPCB/YWHAZ* | *HMBS/PGK1*  *5S/ B-actin* | Adipose tissues  Muscle tissues |
| *Li et al.(2011)[9]* | *H3,B-actin,GAPDH,UBS* and *18S* | *H3/B-actin* | *UBS* | Liver, kidney and thyroid |
|  |  | *GAPDH/B-actin* | *UBS* | Abdominal Fat |
| *Martino et al.(2011)[10]* | *GAPDH, B-actin,B2M,TBP,HPRT-1,PPIA,TOP2B* and *YWHAZ* | *HPRT-1/TBP/PPIA*  *PPIA/GAPDH/ B-actin*  *HPRT-1/TBP/GAPDH* | *TOP2B*  *TOP2B*  *B2M* | Right and left atrium  Right ventricle  Left ventricle |
| *Wang et al.(2011)[11]* | *B-actin,B2M,PGK1,RPG* and *RPS20* | *RPS20/RPG* | *B-actin/B2M/PGK1* | Endometrium |
| *Cinar et al.(2012)[12]* | *B2M,BLM,GAPDH,HPRT1,PPIA,RPL4,SDHA,TBP* and *YWHAZ* | *SDHA/YWHAZ/RPL4* | *GAPDH* | Alveolar macrophages |
| *Zhang et al.(2012)[13]* | *GAPDH, ACTB, H3F3A, HPRT1, RPL32* and *RPS18* | *H3F3A/RPS18*  *PRL32/RPS18* | *GAPDH*  *GAPDH* | Skeletal muscle in Tongcheng pigs  Skeletal muscle in Landrace pigs |
| *Gessner et al.(2013)[14]* | *ATP5G1,SDHA, B-actin,GPI* and *GAPDH* | *ATP5G1*  *SDHA* | *GAPDH*  *RPS9* | Liver  Duodenum |
| *Martinez-Giner et al.(2013)[15]* | *B-actin,B2M,UBC,TBP,YWHAZ,GNB2L1,HMBS,HPRT* and *RPL32* | *TBP/UBC/SDHA*  *TBP/GNB2L1/HPRT1* | *HMBS*  *ACTB/B2M/PGK1* | Ovary  Boar spermatozoa |
| *Zeng et al.(2014)[16]* | *B-actin,B2M,GAPDH,HPRT1,RPL4,SDHA,YWHAZ,PPIA,PGK1,*  *18S* and *BLM* | *GAPDH/RPL4/PPIA* | *BLM/B2M* | Boar spermatozoa |
| *Park et al.(2015)[17]* | *B-actin,ALDOA,B2M,GAPDH,HPAR1,HSPCB,PGK1,POLR2G,*  *PPIA,RPL4,RPS18,SDHA,TBP,TOP2B* and *YWHAZ* | *PPIA/TBP/HSPCB*  *PPIA/TBP/RPL4/RPS18*  *PPIA/TBP* | *HPRT1/SDHA*  *HPRT1/SDHA*  *HPRT1/SDHA* | Liver, lung, kidney, spleen,  stomach, small intestine, and large intestine of Berkshire pigs  Liver, lung, kidney, spleen, stomach, small intestine, and large intestine of Landrace pigs  Liver, lung, kidney, spleen, stomach, small intestine, and large intestine of Duroc pigs |
|  |  | *PPIA/TOP2B/RPL4* | *HPRT1/SDHA* | Liver, lung, kidney, spleen, stomach, small intestine, and large intestine of Yorkshire pigs |
| *Wang et al.(2015)[18]* | *GPX1,PPIA,SDHA,POLR2A, B-actin,AGPAT1,RPL32,H3F3A,*  *GAPDH* and *B2M* | *DRAP1/WSB2* | *B2M/GAPDH* | Skeletal muscle |
| *Li et al.(2016)[19]* | *BANF1,DAK,DPH3,GTF2H3,NSUN5,NUBP1,PRR3,SSU72, TIMM17B,VPS4A,COL1A1,DBI,GAPDH, B-actin,TBP,PPLA, TOP2B,ALDOA,B2M,HPRT1,PGK1,POLR2G,RPL4,RPS18, SDHA,YWHAZ,RPL13A,18S,UXT,TUBA,EIF3K,CLN3* and *RPLP0* | *B-actin/ALDOA/RPS18* | *GAPDH* | *Longissimus dorsi* muscle |
| *Wang et al.(2016)[20]* | *HPRT1,HMBS,18S,B2M* and *B-actin* | *B2M/HMBS/HPRT1* | *18S/ B-actin* | Duodenum |
| *Sandercock et al. (2017)[21]* | *ACTB,SDHA,UBC,B2M,GAPDH* and *eEF-1* | *GAPDH/eEF-1/UBC*  *B-actin/SDHA/UBC* | *ACTB/SDHA*  *GAPDH/eEF-1* | Dorsal root ganglia samples  Spinal cord samples |
| Our study | *5S,RPL4,CANX,ALDOA,PPARGC1A,HSPCB,TBP,*  *B2M,HMBS,HPRT1,B-actin,18S,YWHAZ,RPL32,*  *PGK1,PPIA,TOP2B* and *GAPDH* | *B2M/HMBS/HPRT1* | *GAPDH/18S* | Duodenum, jejunum, ileum and colon |

**Supplementary references:**

1. Erkens T, Van Poucke M, Vandesompele J, Goossens K, Van Zeveren A, Peelman LJ. Development of a new set of reference genes for normalization of real-time RT-PCR data of porcine backfat and longissimus dorsi muscle, and evaluation with PPARGC1A. BMC Biotechnol. 2006;6:41. doi: 10.1186/1472-6750-6-41

2. Kuijk EW, du Puy L, van Tol HT, Haagsman HP, Colenbrander B, Roelen BA. Validation of reference genes for quantitative RT-PCR studies in porcine oocytes and preimplantation embryos. BMC Dev Biol. 2007;7:58. doi: 10.1186/1471-213X-7-58

3. Nygard AB, Jorgensen CB, Cirera S, Fredholm M. Selection of reference genes for gene expression studies in pig tissues using SYBR green qPCR. BMC Mol Biol. 2007;8:67. doi: 10.1186/1471-2199-8-67

4. Svobodová K, Bílek K, Knoll A. Verification of reference genes for relative quantification of gene expression by real-time reverse transcription PCR in the pig. J Appl Genet. 2008;49:263-265. doi: 10.1007/BF03195623

5. Tramontana S, Bionaz M, Sharma A, Graugnard D, Cutler E, Ajmone-Marsan P, et al. Internal controls for quantitative polymerase chain reaction of swine mammary glands during pregnancy and lactation. J Dairy Sci. 2008;91:3057-3066. doi: 10.3168/jds.2008-1164

6. Feng X, Xiong Y, Qian H, Lei M, Xu D, Ren Z. Selection of reference genes for gene expression studies in porcine skeletal muscle using SYBR green qPCR. J Biotechnol. 2010;150: 288-293. doi: 10.1016/j.jbiotec.2010.09.949

7. McBryan J, Hamill RM, Davey G, Lawlor P, Mullen AM. Identification of suitable reference genes for gene expression analysis of pork meat quality and analysis of candidate genes associated with the trait drip loss. Meat Sci. 2010;86:436-439. doi: 10.1016/j.meatsci.2010.05.030

8. Gu Y, Li M, Zhang K, Chen L, Jiang A, Wang JY, et al. Evaluation of endogenous control genes for gene expression studies across multiple tissues and in the specific sets of fat‐and muscle‐type samples of the pig. J Anim Breed Genet. 2011;128:319-325.doi: 10.1111/j.1439-0388.2011.00920.x

9. Li Q, Domig KJ, Ettle T, Windisch W, Mair C, Schedle K. Evaluation of potential reference genes for relative quantification by RT-qPCR in different porcine tissues derived from feeding studies. Int J Mol Sci. 2011;12:1727-1734. doi: 10.3390/ijms12031727

10. Martino A, Cabiati M, Campan M, Prescimone T, Minocci D, Caselli C, et al. Selection of reference genes for normalization of real-time PCR data in minipig heart failure model and evaluation of TNF-alpha mRNA expression. J Biotechnol. 2011;153:92-99. doi: 10.1016/j.jbiotec.2011.04.002

11. Wang S, Li J, Zhang A, Liu M, Zhang H. Selection of reference genes for studies of porcine endometrial gene expression on gestational day 12. Biochem Biophys Res Commun. 2011;408: 265-268. doi: 10.1016/j.bbrc.2011.04.010

12. Cinar MU, Islam MA, Uddin MJ, Tholen E, Tesfaye D, Looft C, et al. Evaluation of suitable reference genes for gene expression studies in porcine alveolar macrophages in response to LPS and LTA. BMC Res Notes. 2012;5:107. doi: 10.1186/1756-0500-5-107

13. Zhang J, Tang Z, Wang N, Long L, Li K. Evaluating a set of reference genes for expression normalization in multiple tissues and skeletal muscle at different development stages in pigs using quantitative real-time polymerase chain reaction. DNA Cell Biol. 2012;31:106-113. doi: 10.1089/dna.2011.1249

14. Gessner DK, Fiesel A, Most E, Dinges J, Wen G, Ringseis R, et al. Supplementation of a grape seed and grape marc meal extract decreases activities of the oxidative stress-responsive transcription factors NF-κB and Nrf2 in the duodenal mucosa of pigs. Acta Vet Scand. 2013;55:18. doi: 10.1186/1751-0147-55-18

15. Martinez-Giner M, Noguera JL, Balcells I, Fernandez-Rodriguez A, Pena RN. Selection of internal control genes for real-time quantitative PCR in ovary and uterus of sows across pregnancy. PLoS One. 2013;8:e66023. doi: 10.1371/journal.pone.0066023

16. Zeng C, He L, Peng W, Ding L, Tang K, Fang D, et al. Selection of optimal reference genes for quantitative RT-PCR studies of boar spermatozoa cryopreservation. Cryobiology. 2014;68:113-121. doi: 10.1016/j.cryobiol.2014.01.004

17. Park S-J, Kwon SG, Hwang JH, Park DH, Kim TW, Kim CW, et al. Selection of appropriate reference genes for RT-qPCR analysis in Berkshire, Duroc, Landrace, and Yorkshire pigs. Gene.2015; 558:152-158. doi: 10.1016/j.gene.2014.12.052

18. Wang Y, Zhao Y, Li J, Liu H, Ernst CW, Liu X, et al. Evaluation of housekeeping genes for normalizing real-time quantitative PCR assays in pig skeletal muscle at multiple developmental stages. Gene. 2015; 565:235-241. doi: 10.1016/j.gene.2015.04.016

19. Li X, Huang K, Chen F, Li W, Sun S, Shi XE, et al. Verification of suitable and reliable reference genes for quantitative real-time PCR during adipogenic differentiation in porcine intramuscular stromal-vascular cells. Animal. 2016;10:947-952. doi: 10.1017/S1751731115002748

20. Wang S, Guo C, Zhou L, Zhong Z, Zhu W, Huang Y, et al. Effects of dietary supplementation with epidermal growth factor-expressing *Saccharomyces cerevisiae* on duodenal development in weaned piglets. Br J Nutr. 2016;115:1509-1520. doi: 10.1017/S0007114516000738

21. Sandercock DA, Coe JE, Di Giminiani P, Edwards SA. Determination of stable reference genes for RT-qPCR expression data in mechanistic pain studies on pig dorsal root ganglia and spinal cord. Res Vet Sci. 2017;114:493-501. doi: 10.1016/j.rvsc.2017.09.025
