# Supplementary material for: A new set of reference housekeeping genes for the normalization RT-qPCR data from the intestine of piglets during weaning
Source: PLoS One. 2018 Sep 26;13(9):e0204583. doi: 10.1371/journal.pone.0204583 (PMC6157878; doi:10.1371/journal.pone.0204583)

**S1 Fig. Identification of PCR fragments amplified using gene-specific primers for the reference genes tested.** Agarose gel (2%) electrophoresis shows a band at the expected size for each reference gene. M: DNA Marker I (MD101) (100–600 bp), 1: *HMBS*, 2: *ALP*, 3: *18S*, 4: *TBP*, 5: *RPL19*, 6: *PPARGC1A*, 7: *5S*, 8: *CANX*, 9: *HSPCB*, 10: *B-actin*, 11: *UBC*, 12: *B2M*, 13: *HPRT1*, 14: *PGK1*, 15: *YWHAZ*, 16: *GAPDH*, 17: *PPIA*, 18: *RPL32*, and 19: *ALDOA*.


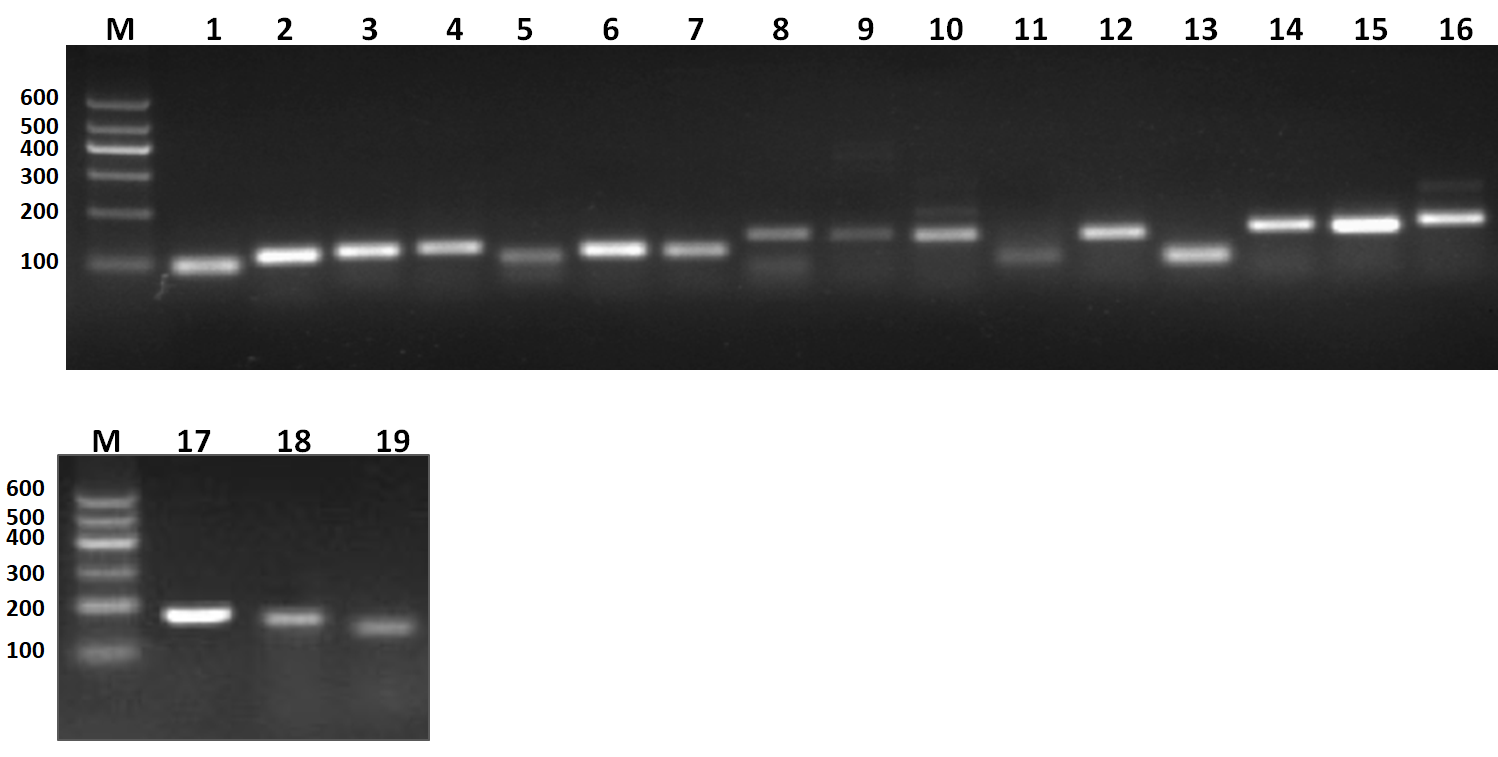

Supplement: S1 Fig — Agarose gel (2%) electrophoresis shows a band at the expected size for each reference gene. M: DNA Marker I (MD101) (100–600 bp), 1: HMBS, 2: ALP, 3: 18S, 4: TBP, 5: RPL19, 6: PPARGC1A, 7: 5S, 8: CANX, 9: HSPCB, 10: B-actin, 11: UBC, 12: B2M, 13: HPRT1, 14: PGK1, 15: YWHAZ, 16: GAPDH, 17: PPIA, 18: RPL32, and 19: ALDOA. (DOCX) [file pone.0204583.s009.docx]
