# Supplementary material for: A new set of reference housekeeping genes for the normalization RT-qPCR data from the intestine of piglets during weaning
Source: PLoS One. 2018 Sep 26;13(9):e0204583. doi: 10.1371/journal.pone.0204583 (PMC6157878; doi:10.1371/journal.pone.0204583)
Supplement: S2 Fig — (DOCX) [file pone.0204583.s010.docx]

**S2 Fig. Melting curves and standard curves of eighteen reference genes and a target gene.**


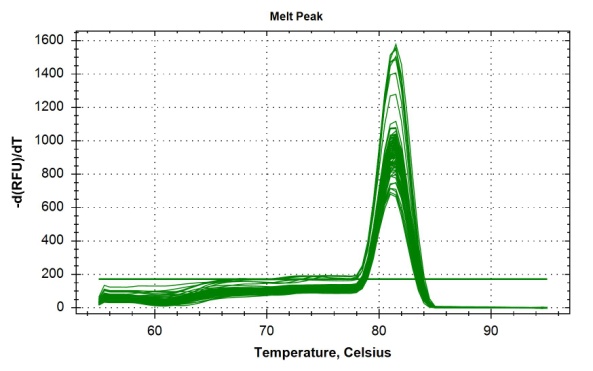

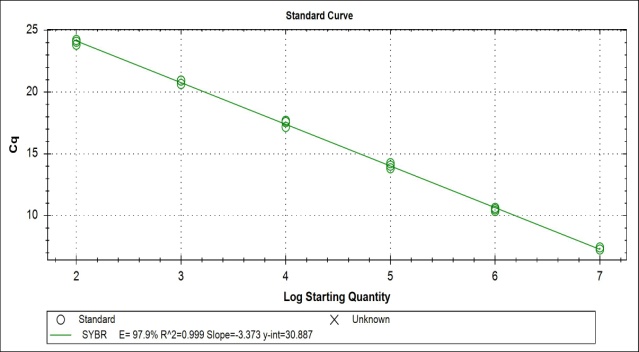


S2 Fig A. Melting curve & standard curve of *YWHA*


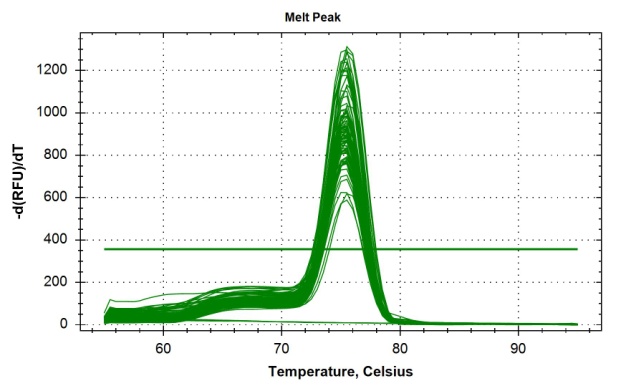

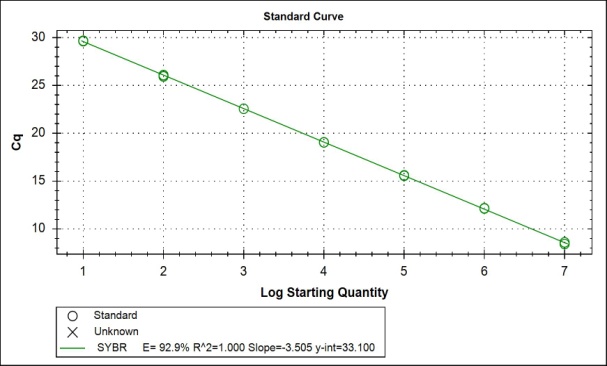


S2 Fig B. Melting curve & standard curve of *UBC*


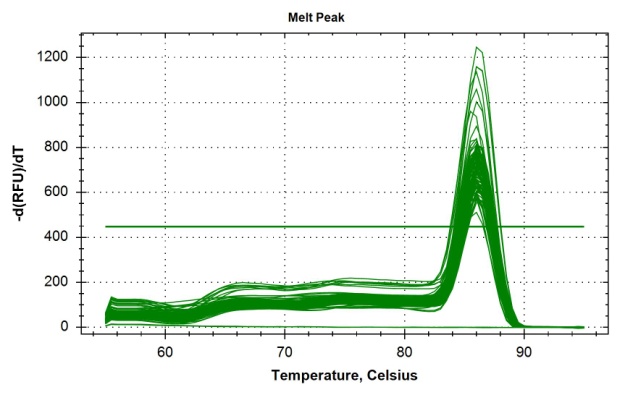

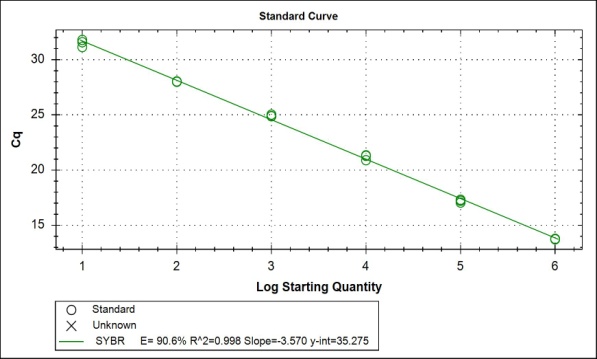


S2 Fig C. Melting curve & standard curve of *TBP*


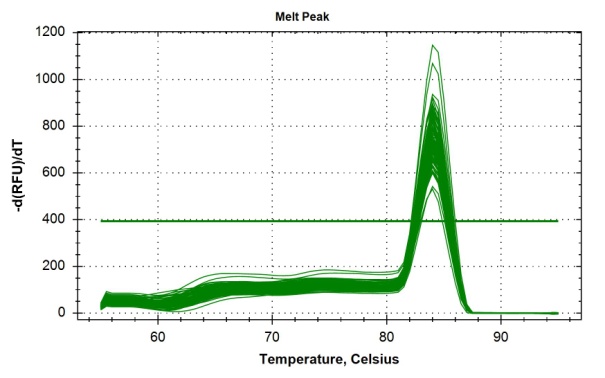

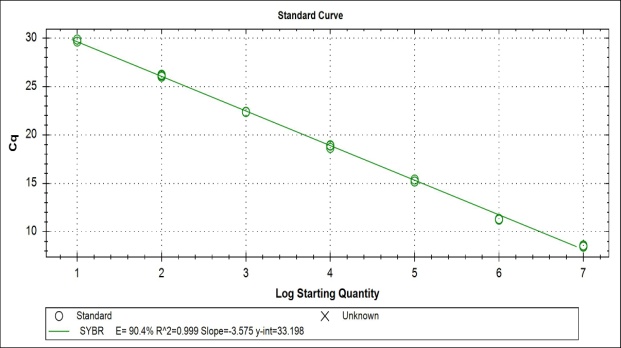


S2 Fig D. Melting curve & standard curve of *RPL32*


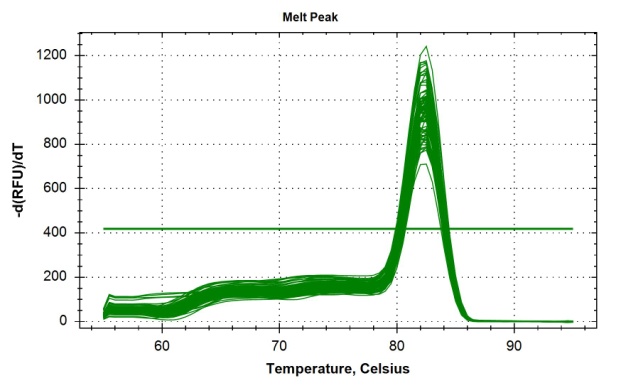

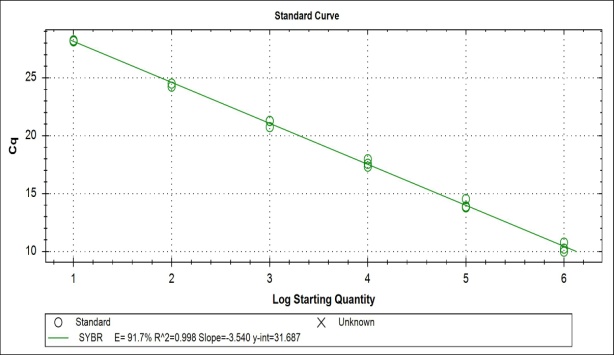


S2 Fig E. Melting curve & standard curve of *RPL19*


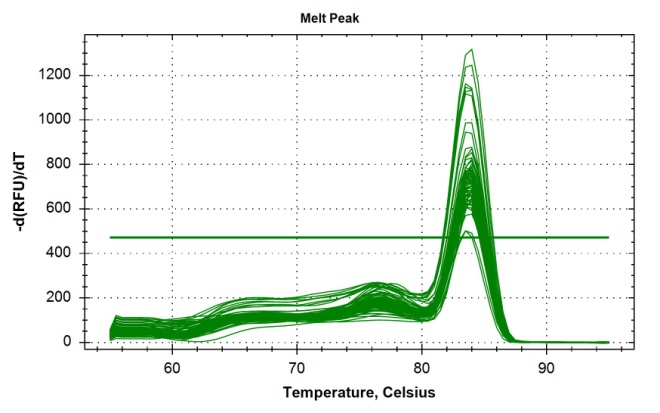

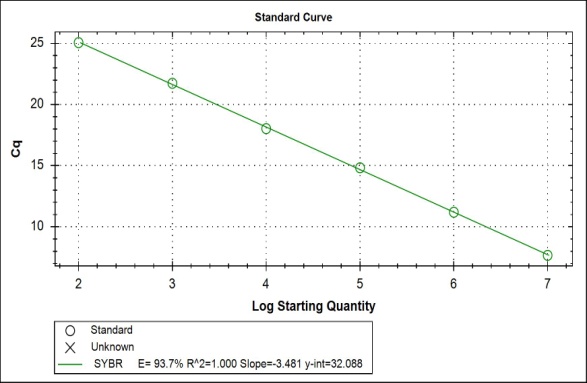


S2 Fig F. Melting curve & standard curve of *PPARGGIA*


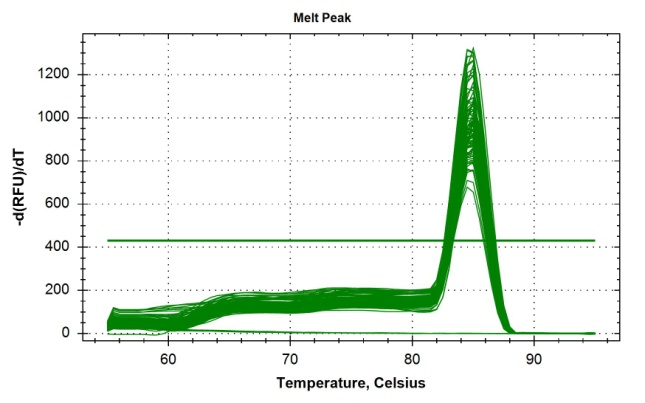

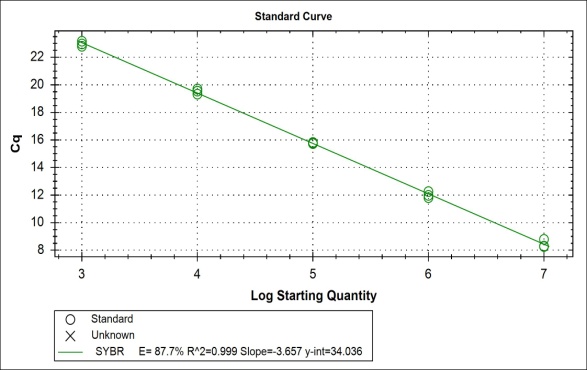


S2 Fig G. Melting curve & standard curve of *PGK11*


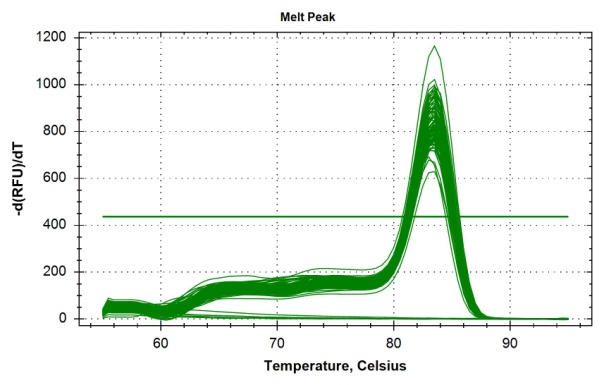

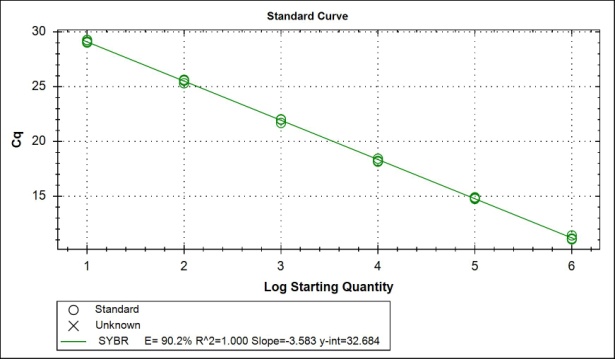


S2 Fig H. Melting curve & standard curve of *HSPCB*


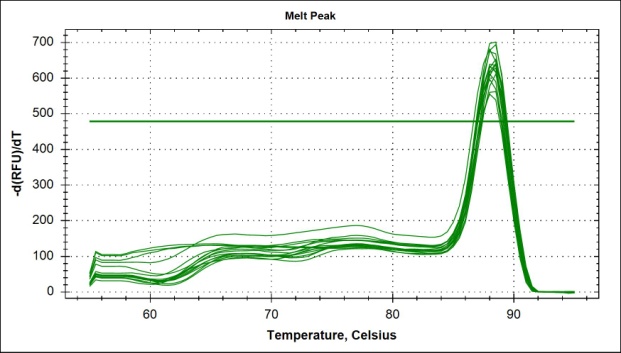

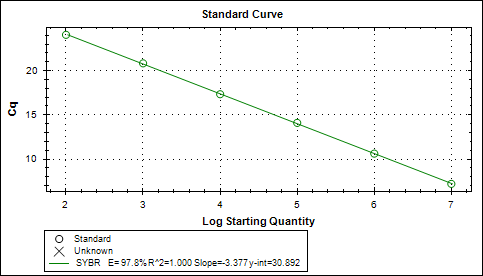


S2 Fig J. Melting curve & standard curve of *CANX*


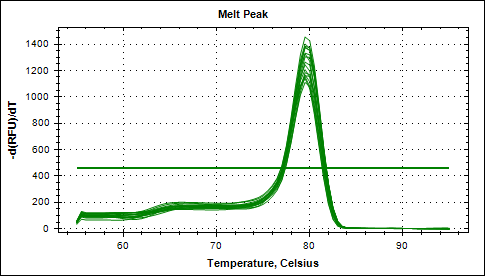

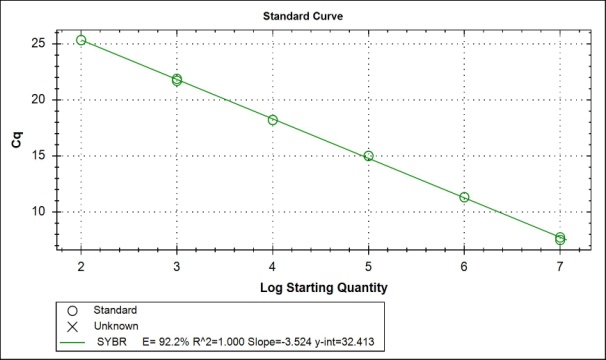


S2 Fig K. Melting curve & standard curve of *ALDOA*


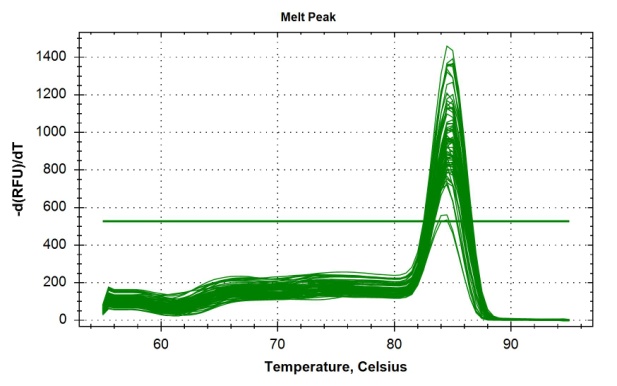

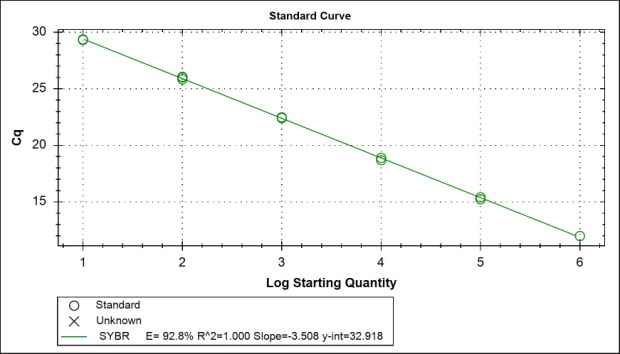


S2 Fig L. Melting curve & standard curve of *5S*


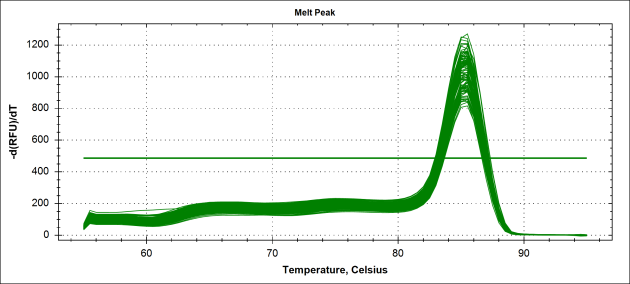

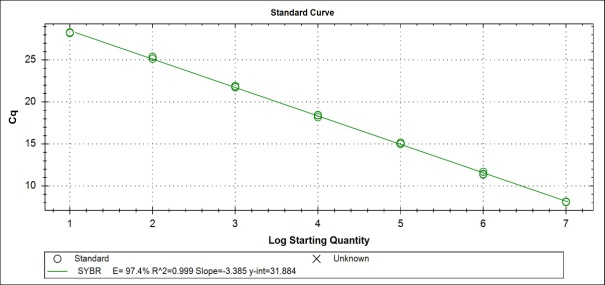


S2 Fig M. Melting curve & standard curve of *18S*


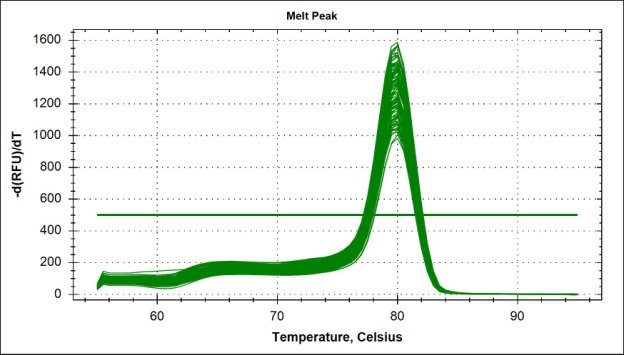

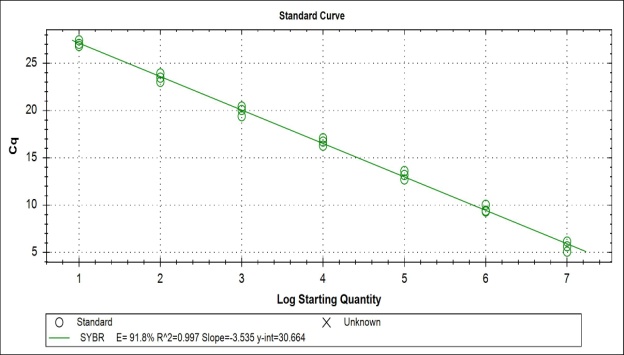


S2 Fig N. Melting curve & standard curve of *B2M*


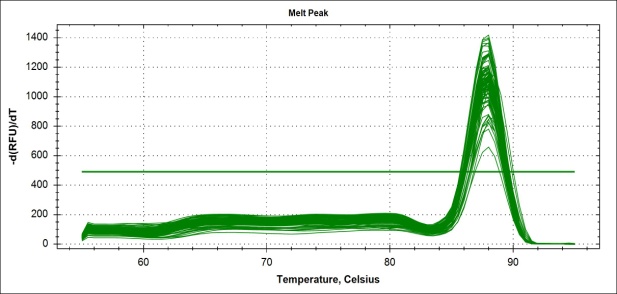

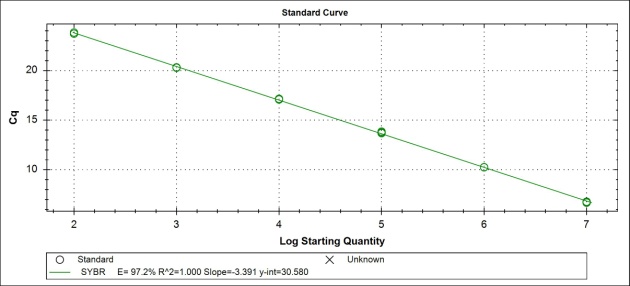


S2 Fig O. Melting curve & standard curve of *B-actin*


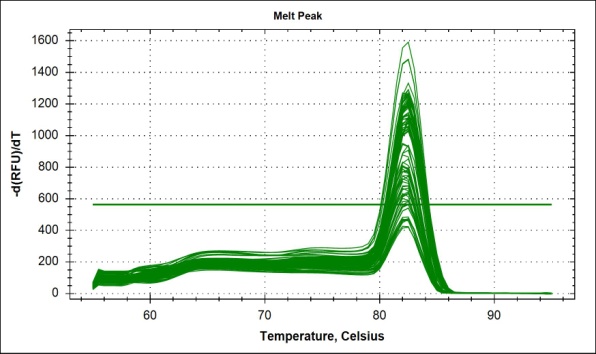

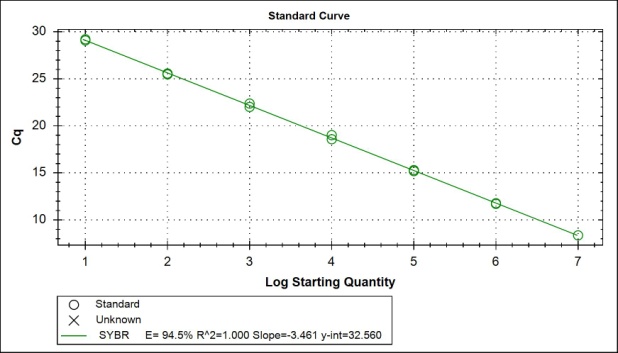


S2 Fig P. Melting curve & standard curve of *GAPDH*


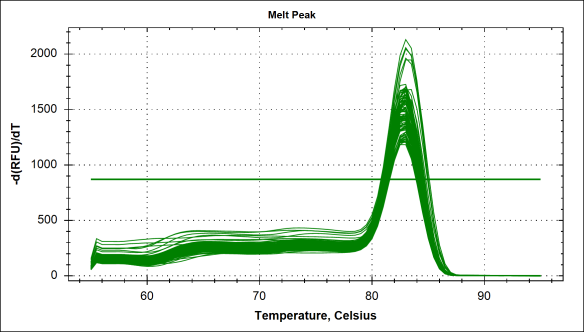

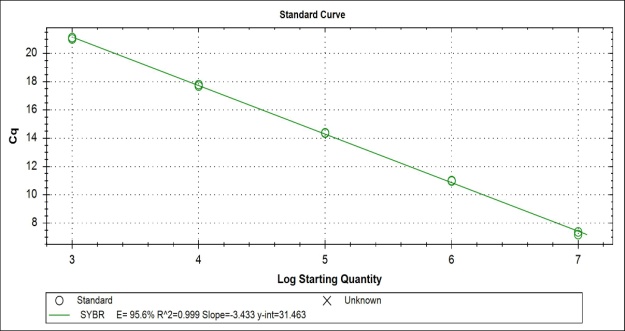


S2 Fig Q. Melting curve & standard curve of *HMBS*


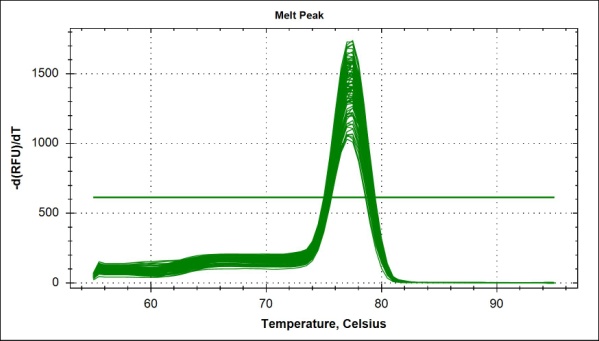

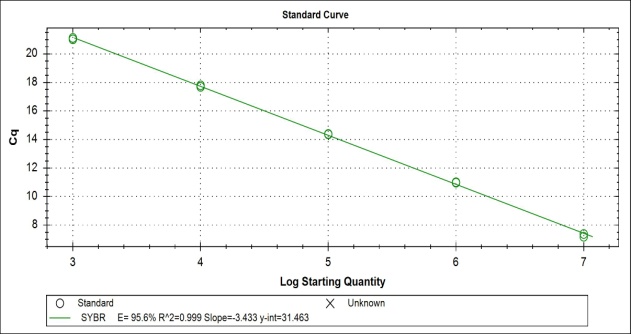


S2 Fig R. Melting curve & standard curve of *HPRT1*


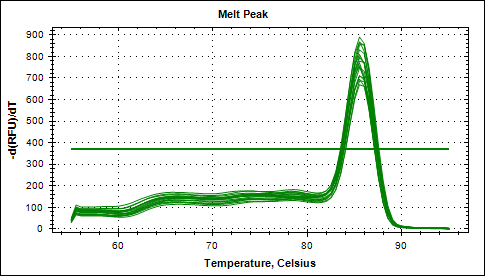

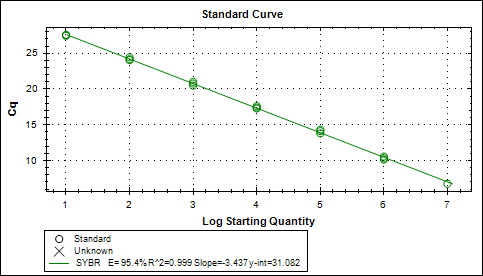


S2 Fig X. Melting curve & standard curve of *ALP*
