# Supplementary material for: A new set of reference housekeeping genes for the normalization RT-qPCR data from the intestine of piglets during weaning
Source: PLoS One. 2018 Sep 26;13(9):e0204583. doi: 10.1371/journal.pone.0204583 (PMC6157878; doi:10.1371/journal.pone.0204583)
Supplement: S6 Fig — A) Rank order of gene expression stability based on average expression stability values (M) for the reference genes from least stable (left) to most stable (right). B) Pairwise variation analysis (V) to determine the optimal number of reference genes for RT-qPCR data normalization. (DOCX) [file pone.0204583.s014.docx]

**S6 Fig. Gene expression stability and rankings of reference genes at 21 days post-weaning as calculated by GeNorm.**

A) Rank order of gene expression stability based on average expression stability values (M) for the reference genes from least stable (left) to most stable (right).

B) Pairwise variation analysis (V) to determine the optimal number of reference genes for RT-qPCR data normalization.
